# The unexploited potential of data systems tracking medicines utilization: an opportunity to improve access to oncology combination therapies

**DOI:** 10.3389/fphar.2025.1532022

**Published:** 2025-08-20

**Authors:** Steven Simoens, David Dodwell, Michael Hartevelt, Peter Lindgren, Michele Pistollato, Lisa G. Pont, Caridad Pontes, Alexander Roediger, Antun Sablek, Eric Van Ganse, Qinyi Wang, Chris Wenger, Tim Wilsdon, Entela Xoxi, Brian Godman

**Affiliations:** ^1^ KU Leuven Department of Pharmaceutical and Pharmacological Sciences, Leuven, Belgium; ^2^ Oxford Population Health, University of Oxford, Oxford, United Kingdom; ^3^ Global Oncology Policy, Merck, Rahway, NJ, United States; ^4^ Swedish Institute for Health Economics, Lund, Sweden; ^5^ Health Economics, Karolinska Institute, Solna, Sweden; ^6^ Charles River Associates, London, United Kingdom; ^7^ Graduate School of Health, University of Technology Sydney, Sydney, NSW, Australia; ^8^ Department of Pharmacology, Therapeutics and Toxicology, Autonomous University of Barcelona, Barcelona, Spain; ^9^ Charles River Associates, Brussels, Belgium; ^10^ Pharmacoepidemiology Unit, Claude Bernard University Lyon 1, Lyon, France; ^11^ Charles River Associates, Cambridge, United Kingdom; ^12^ Aquantic AG, Zeiningen, Switzerland; ^13^ Postgraduate School of Health Economics and Management (ALTEMS), Catholic University of the Sacred Heart, Rome, Italy; ^14^ Strathclyde Institute of Pharmacy and Biomedical Sciences, University of Strathclyde, Glasgow, United Kingdom; ^15^ Department of Public Health Pharmacy and Management, School of Pharmacy, Sefako Makgatho Health Sciences University, Pretoria, South Africa

**Keywords:** oncology combination therapies, individual patient-level healthcare data, utilization tracking, pricing and reimbursement, pharmaceutical policy, Europe, Australia

## Abstract

**Background:**

While progress has been made in oncology treatments, including the introduction of combination therapies, barriers affect patient access. There are approaches that could improve access including combination-specific pricing that allow the price to reflect whether a product is used in monotherapy or in combination. The feasibility of this solution requires data on the utilization of combination therapies.

**Aim:**

To investigate the ability of data systems across Belgium, England, France, Italy, Spain, Sweden, Switzerland and Australia to track drug utilization of combination therapies, particularly in oncology.

**Methods:**

A targeted literature review was conducted to investigate the attributes of the key data systems. One-on-one semi-structured interviews were subsequently conducted with country experts to validate the research, who were screened based on their expertise and knowledge of the respective data and reimbursement systems in their countries, followed by an advisory board to align on policy recommendations. Country-specific and cross-border insights were gathered from nine experts across these countries.

**Results:**

There are data systems that routinely collect medicine utilization data across seven European countries and Australia. These datasets can potentially be leveraged to track the utilization of combination therapies. Where available, administrative data systems, such as reimbursement claims data, can be leveraged, though other types of systems, such as product registries, may be more suitable in some countries, emphasizing the need to consider country-specific nuances. Using existing data systems is likely to be less resource-intensive than setting up a novel system for this application. While viable sources of data exist in most countries, many need improving to fully harness their tracking potential. There are several common areas where improvement is needed to track combination therapies effectively. These include data quality, access for different stakeholders, minimizing the burden of data entry and management, and increasing support from national authorities to foster multi-stakeholder engagement.

**Conclusion:**

While most countries possess data systems that could serve as a foundation for tracking combination oncology therapies, these systems require optimization and proper implementation. Our core recommendation is for policymakers to explore the expansion and enhancement of data infrastructures.

## 1 Introduction

Healthcare systems are under pressure from the growing and ageing population, as well as the rising burden of chronic illnesses, especially cancer (Sung et al., 2021). New solutions are needed to ensure novel treatments can be made widely accessible, to promote better patient care, within limited healthcare budgets (Bajwa et al., 2021). In this context, the digitalization and greater availability and use of data in healthcare can deliver several benefits across therapy areas and help countries achieve better, sustainable and more cost-effective care (Bajwa et al., 2021; Pisana et al., 2022). Routinely collected data on the utilization of medicines can promote rational and appropriate use of evidence-based therapies according to clinical guidelines for the benefit of patients, reduce treatment heterogeneity across patients, improve equitable access to innovative medicines by supporting novel payment models, and optimize resource allocation for pharmaceutical spending (Afify, 2016; OECD, 2019; Hofmarcher et al., 2022; Pisana et al., 2022).

Decision-makers have identified the critical role of digital healthcare transformation, and the value of health data in general, and have begun to emphasize this in their agendas. For example, the European Commission, acknowledging the necessity for enhancing the healthcare data landscape, has initiated steps towards creating a more unified and efficient system. The introduction of the European Health Data Space (EHDS) aims to standardize data practices, infrastructure, and governance across the EU (European Health Data Space, 2024). At a country level, the establishment of Health Data Authorities in a number of countries, including Belgium and Finland, and the Health Data Hub in France reinforces the recognition that building healthcare data capabilities is critical in the current landscape (Ayora, 2024; Boland and Roy, 2024).

However, there are gaps and challenges with existing healthcare data, including medicine utilization data. This means they cannot yet be leveraged optimally to derive benefits for patients and healthcare systems (OECD, 2019; Pisana et al., 2022; Oderkirk, 2021). Challenges include siloed data systems that cannot be linked together, limitations around data quality and validity, limited coverage (e.g., hospital medicines not covered), data input inefficiencies (e.g., various platforms for data collection resulting in data duplication), limitations on access to collected data due to data security and privacy concerns, and a lack of centralized support from decision makers (OECD, 2019; Martins, 2021; Pisana et al., 2022; Mills and Kanavos, 2023). There is, therefore, a pressing need to improve current data systems and reduce the burden placed on individuals in charge of data input, especially healthcare professionals.

The value of healthcare data that can track and improve the use of oncology treatments will only grow. The likely increase in spending and the need to use the limited resources effectively in response to the increasing prevalence and innovation in this high-priority disease area are key considerations for decision-makers. Combination therapies, treatments made up of two or more “constituents”, will play an increasingly important role in addressing cancer patients’ needs and improving prognosis compared to monotherapies (Zimmermann et al., 2007; Jardim et al., 2020; Chyuan et al., 2021; Yang et al., 2022). However, research and experience have shown that reimbursement of, and patient access to, these treatments is a concern in Europe and in other countries such as Australia and Canada because current reimbursement systems struggle to distinguish when a medicine is provided as a monotherapy or as a constituent of a combination (Latimer et al., 2021; Pistollato et al., 2021; Rogoza, Husereau and Millson, 2022). For instance, combination therapies in Europe take an average of 193 days longer to become available compared to all oncology products, and they are less frequently reimbursed compared with monotherapies (Pistollato et al., 2023; Wilsdon and Morlotti, 2022). With a growing pipeline and the expected launch of 55 novel combination therapies in Europe in the next 5 years, there is an urgent need to ameliorate their access challenges (Albrecht et al., 2020; IQVIA Institute for Human Data Science, 2023; Pistollato et al., 2023). One potential strategy to improve access to combination therapies is to implement pricing models that specifically align with the value these therapies provide (EFPIA, 2023; Pistollato et al., 2023). Combination-specific pricing allows the price to reflect whether a product is used in monotherapy or combination (Preckler and Espín, 2022; Wilsdon and Morlotti, 2022). Combination-specific pricing requires tracking systems capable of distinguishing between use in monotherapy and as a combination, to support informed pricing decisions and ensure accurate rebate calculations for combination therapies where the need arises, including managed entry agreements. The lack of sophisticated data systems and the necessary infrastructure for tracking utilization of cancer medicines currently stand as major challenges to implementing these innovative solutions (EFPIA, 2020; Pisana et al., 2022). Enhancing data systems to provide policymakers with better information on utilization of medicines, including combination therapies, could help improve the utilization of available resources for oncology medicines and ensure that a broader range of patients has access to proven effective medicines, thereby improving the care of patients with cancer. This will benefit all key stakeholders.

To date, limited research has been undertaken on the availability and robustness of medicine utilization tracking systems for combination therapies, and how they are implemented. This analysis aims to fill these knowledge gaps by describing the current state of data capabilities for combination therapies across seven European countries (Belgium, England, France, Italy, Spain, Sweden and Switzerland) and Australia to identify challenges and best practices in tracking medicine utilization. We focus on oncology medicines in the research, but the benefits of medicine utilization tracking systems apply more broadly. We finally derive the minimum elements of data systems required for routine tracking of combination therapy utilization and translate these into policy recommendations for implementing a tracking system.

## 2 Materials and methods

### 2.1 Selection of countries

The geographic scope included Australia and seven European countries (Belgium, England, France, Italy, Spain, Sweden and Switzerland). Countries were selected based on the presence of existing data systems containing routinely collected data (OECD, 2019). The selected range of countries also includes a range of different healthcare systems enabling the exploration of how different healthcare data environments and infrastructures affect the use of the different data systems and the ability to monitor drug utilization. In addition, this enables exploring the use of various data systems to support decision-making and policies as well as data management and access practices (OECD, 2019; Latimer et al., 2021; EFPIA, 2023).

### 2.2 Study framework

Several key research questions were developed with the help of experts to guide the research into medicine utilization tracking systems and their application. As a result, this ensures that the necessary insights would be captured to shape the policy recommendations. The key research questions included:

• Are there existing data tracking systems in each country, and what information do they track?
• To what extent are existing data systems capable of routine tracking of medicine utilization?
• How effective are the existing data systems in capturing medicine utilization in the context of oncology combination therapies?
• Hence, what are the minimum elements/components required for tracking medicine utilization (particularly oncology combination therapies)?

### 2.3 Targeted literature review

We initially screened major publications and reports on health data systems, leveraging Pisana et al., 2022 and the OECD report “Using Routinely Collected Data to Inform Pharmaceutical Policies: Analytical Report for OECD, 2019, as well as related references. These sources helped us identify which countries have data systems to monitor the use of medicines, providing a foundation for selecting countries to include in our study. Based on this initial review, we focused on seven European countries–Belgium, England, France, Italy, Spain, Sweden, and Switzerland–along with Australia, each of which has relevant data systems in place.

We then conducted a targeted literature review in each country to verify the relevance of the shortlisted data systems for medicine utilization tracking and to understand their key features. The review leveraged 65 sources, which included peer-reviewed articles and the grey literature (e.g., governmental official sources such as health data system official websites and white papers from international organizations). Search engines included PubMed, Google Scholar and local Google sites, focusing on documents published between 2019 and 2023 written in English or local languages. The following search terms were used: “(country)”, “Europe”, “oncology”, “cancer medicines”, “combination therapies”, “availability”, “healthcare data”, “data systems”, “data infrastructure”, “registries”, “reimbursement claims”, “prescription record”, “electronic health records”, “medical records”, “medicine utilization”, “monitoring”, “tracking utilization”, “routinely collected”, “scope”, “patient-level”, “data access”, “transparency”, “quality”, “data management”, “integration”, “funding”, “stakeholders”, “reimbursement and access”, “policies”, and “application”.

Based on the targeted literature review, we identified the most relevant data system(s) per country for further analysis (Table 1). Eleven data systems were identified, one per country except in Belgium, England, and Spain with two relevant data systems for each.

**TABLE 1:** Type and identity of data systems used for analysis of country capabilities in tracking utilization of oncology combination therapies.

Country	Data source used for analysis	Type of data system
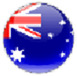	Pharmaceutical Benefits Scheme (PBS) data system	Reimbursement claims data
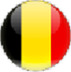	Belgian inter-mutual agency (IMA-AIM) data system	Reimbursement claims data
	Chapter IV ‘intention to treat’ data	Physician prescription request record
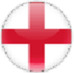	Blueteq forms	Physician prescription request record
	Systemic anti-cancer therapy (SACT) data system	Registries
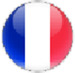	French hospital discharge (PMSI) data system	Reimbursement claims data
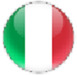	Italian medicines agency (AIFA) registries	Product registries
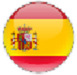	Catalan health data system	Medical records and reimbursement claims data
	Valtermed	Disease registries
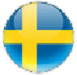	National quality registries for cancer on the information network for cancer care (INCA), supported by regional care centers (RCC)	Cancer registries
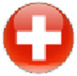	SmartMIP data	Reimbursement claims data

Sources: Australia – https://www.pbs.gov.au/pbs/home; Belgium IMA-AIM – https://www.ima-aim.be/; Belgium Chapter IV –; England Blueteq – https://blueteq.com/commissioner-high-cost-drugs-new/; England SACT – http://www.chemodataset.nhs.uk/home; France – https://www.snds.gouv.fr/SNDS/Accueil; Italy – https://www.aifa.gov.it/registri-farmaci-sottoposti-a-monitoraggio; Spain Catalan–Roig Izquierdo et al. (2020); Spain Valtermed – https://www.sanidad.gob.es/areas/farmacia/infoMedicamentos/valtermed/; Sweden – https://cancercentrum.se/samverkan/vara-uppdrag/kunskapsstyrning/kvalitetsregister/stod-for-kvalitetsregister/om-inloggning/om-inca/; Switzerland – https://aquantic.ch/produkte/smartmip/#:∼:text=Mit%20SmartMIP%20haben%20Sie%20ein%20Tool,%20das%20Ihnen%20einen%20standardisierten

### 2.4 Qualitative analysis of the data systems

To assess the current state of data systems across the countries in scope, the research team conducted a qualitative analysis based on findings from the targeted literature review of their current abilities to track utilization of combination therapies and their potential to support combination-specific pricing. The data systems were compared based on a range of dimensions agreed with the experts: the source of data, the scope, the granularity of the data, the ability to access the data and their transparency, the quality of the data, the data management and integration, the funding, and the stakeholders involved. Each system was given a rating of “low”, “medium” or “high”, where “high” indicates the fewest changes will be required to allow for combination therapy tracking.

### 2.5 Expert interviews

Individual 1-h semi-structured interviews in English with nine country experts were conducted between March 2023 and August 2023 using a structured interview guide (Table 2) to validate the literature review findings and the assessment of the data system capabilities, and also to better understand details not identified in the literature. The same interviewers conducted all interviews to standardize the interviewing style and quality. The experts were selected based on their academic contributions to the field of health data systems and their experience within the relevant countries, and criteria were further refined to identify those with specialized knowledge in oncology treatment tracking and usage.

**TABLE 2:** Cross-country semi-structured interview guide.

Interview guide
1. What do you think are the main challenges for implementing a tracking system for combination use of medicines to treat patients with cancer in European countries/your country? 2. How important do you think it is to have data infrastructure to track usage of combination therapies? 3. What type of data is the most useful for tracking the use of combination therapies in your country (reimbursement claims data, patient/product/disease registries, EHRs, etc.)? a. In your experience, what are the advantages and disadvantages of each type of data? b. Are there any challenges in obtaining and using these types of data for tracking combination therapies? c. Do you see any potential ethical or privacy concerns related to the use of patient data for tracking combination therapies? 4. Our initial research shows that some countries use reimbursement claims data. How reliable are these data sources, and are there any potential biases to consider? a. Are there specific challenges if oncology combinations are only used in hospitals, given that some European reimbursement agencies only handle ambulatory care medicines (e.g., TLV in Sweden)? b. Are there any challenges in accessing these data sources, or is the data readily available? c. Are there any limitations to using only reimbursement claims data for tracking combination therapies? 5. What is your opinion on the potential benefits of tracking utilization of combination therapies, such as combination-specific pricing or indication-based pricing? 6. To what extent do you think payers would be supportive of establishing a tracking system for combination use? Do you anticipate any resistance or challenges in implementing such a system? a. In your opinion, what are the main factors influencing payers’/health authorities’ attitudes towards a tracking system for combination use? b. What funding do you think would be needed for the implementation of a tracking system for combination use, and how should funding be obtained? c. What potential challenges or barriers should be addressed in order to gain support from payers/health authorities? 7. Do you think that physicians and healthcare professionals would be willing to record usage of combination therapies, or would this add excessive administrative burden? a. How could collection of combination therapy usage be linked to other information collected in hospitals? b. What opportunities are there, if any, to streamline data collection to reduce burden on healthcare professionals? 8. What kind of tracking system do you think would be most beneficial and feasible to implement in the context of combination therapies? 9. How important is it to integrate different recording methods across regions or hospitals, especially in Italy, Spain, Sweden and Switzerland? 10. Who do you think should be involved in the implementation of a tracking system for combination use, and what should be their role? a. What do you think is the role of the pharmaceutical industry in supporting the implementation of a tracking system for combination use? b. How will data privacy be protected? 11. Based on your experience and perspective, what the minimal requirements that are needed for an efficient tracking system for oncology combination therapies? a. Are there any existing examples or best practices that could serve as models for establishing policy recommendations?

### 2.6 Advisory board meeting

To systematize and operationalize the findings from the primary and secondary research, a 3-h advisory board was held involving seven of the nine country experts interviewed. During the advisory board, experts aligned on the (relative) capability ratings across countries and the minimum necessary components a data system needs to capture to allow effective tracking of oncology combination therapies. Policy recommendations were collaboratively developed and aligned on. As two experts were unable to join the advisory board, two additional 1-h interviews were held to capture their feedback. Their feedback was subsequently integrated during the development of the final recommendations. All country experts have subsequently been invited to collaborate to the development of this article, which reflects the output from the research process, and agreed to be co-authors.

## 3 Results

In this section, we present and analyze the comparative capabilities of data systems across different countries, focusing on their potential to track combination oncology therapies. By examining the strengths and weaknesses of these systems, we aim to identify key challenges that hinder effective tracking of combination treatments. Following this comparative analysis, we provide a set of recommendations to address the identified challenges. These recommendations are structured in two parts: the first focuses on minimizing the resources required to optimize the existing tracking infrastructure, while the second outlines the minimum data requirements that data systems should collect to support combination-specific pricing models.

### 3.1 Comparison of capabilities of data systems between countries with potential for tracking combination oncology therapies

Across the eight countries in scope, all have one or more potential data systems that can be used to track medicine utilization.

Table 3 shows considerable variation between data systems across the parameters assessed, considering they employ distinct data sources that vary in their objectives for data collection. Detailed analyses of each country are available in the Supplementary Material (Supplementary Tables S1-S8). Further analyses of the data systems show that their capability for tracking the use of combination therapies differs substantially across the studied countries (Table 4), which could well be driven by inherent differences between data sources or data infrastructure. For instance, Australia’s Pharmaceutical Benefits Scheme (PBS) data system is a robust source of data for tracking the utilization of oncology combination therapies as it can fully differentiate monotherapies and combinations with national coverage. This robust capability may result from the recording of indication-based pricing, i.e., for medicines with multiple indications, prices are negotiated individually for each indication (different from the published price) based on weightings from estimated drug utilization (Department of Health and Aged Care, 2017). On the other end of the spectrum, Sweden lacks a national data system for hospital medicines (it only has a data system for retail drugs), and the regional cancer registries available typically have inferior coverage as data entry is optional and, therefore, sporadic. Overall, existing data systems will require different adjustments and additions to achieve robust tracking of the utilization of combination therapies, taking into account the best practices and challenges identified from country analyses.

**TABLE 3:** High-level summary of the characteristics of relevant data systems identified in selected countries.

*Name of data system*	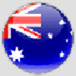	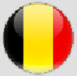	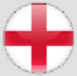	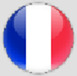	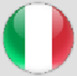	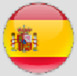	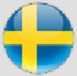	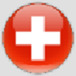
	PBS	IMA-AIM and Chapter IV	Blueteq and SACT	PMSI	AIFA registries	Catalan health and valtermed	Inca, RCC	SmartMIP data
*Data source*	Reimbursement claims data	Reimbursement claims data and Physician prescription request record	Physician prescription request record and Registry	Reimbursement claims data	Product registries	Medical records and reimbursement claims data and Disease registries	Various cancer registries	Reimbursement claims data
*Scope*	All reimbursed drugs, including oncology combinations	IMA-AIM data captures all drugs, including innovative drugs under Chapter IV that require reimbursement authorization	Blueteq covers cancer drugs under the CDF; SACT dataset tracks most oncology drugs	All high-cost drugs on the *liste-en-sus* (ex-DRG) are included	Mainly innovative drugs recognized by AIFA (though AIFA ‘innovation’ status is not always required)	Catalan Health data system covers outpatient drugs dispensed from hospitals (includes most oncology drugs); Valtermed only applies to drugs with high uncertainty (e.g., rare diseases)	Since there are no data systems tracking utilization of hospital medicines, only cancer registries can be considered (the Swedish Prescribed Drug Registry only tracks ex-hospital outpatient drugs)	Drugs included in Art. 71 and specialty medicines
*Granularity*	Individual codes exist for each indication, including separate codes for combinations	IMA-AIM data does not capture indications, Chapter IV data does	Both Blueteq and SACT track the indication (though the indication may not always be clear)	Indications are recorded for drugs on the *liste-en-sus*	Data is very granular – includes the diagnosis, indication, line of treatment, number of packs, treatment duration, use as monotherapy or in combinationetc.	Catalan data includes patient-level data on indication and treatment; Valtermed also captures usage by indication	Registries capture the indication	Indication codes are recorded by HCPs but not in sick funds, so algorithms are used to extrapolate/deduce the code
*Data access/transparency*	De-identified data is publicly available – stakeholders (including pharma companies) can get a monthly report on utilization by indication	Payers and third parties (e.g., industry) can access both reimbursement authorization and IMA-AIM data (industry access is only for their own products)	SACT can be accessed by the MoH and NICE (and pharma companies) at cost and after ethics review; Blueteq data under the CDF is not captured in SACT and is available for certain third parties (e.g., researchers), but cannot be accessed by industry	Several administrations have direct and permanent access to PMSI data under conditions that guarantee anonymity; data can be requested by other public or private actors for research projects (e.g., through data vendors or CROs)	AIFA has access to the data; pharma companies have access registries for their own product(s) at any time	For both systems, data cannot be accessed for commercial purposes, but can be accessed by HCPs/hospitals and reimbursement agencies/public services	Data can only be accessed for research purposes after ethics approval	Pharma companies can access aggregated data on their own products
*Data quality*	Robust – automated data collection (e.g., via e-prescriptions), periodic audits; little delay to access updated data	Limited – no audits; 7–9 months of delay in access to updated data	Relatively high, but stage/morphology of disease is often incomplete in SACT	There are some reservations on the quality of data on the indication; 6–9 months of delay	Limited – no audits; up to 1 year delay	In Catalan, quality check systems are in place, and hospitals are required to collect data due to the contracting system and can be inspected; Valtermed is based on RWD so there may be minor issues	Limited – manual reporting, low reporting rate by physicians	Limited – data quality varies significantly between sick funds and no robust audits are performed; months/years of delay
*Data management*	Physicians select the correct code for a therapy (includes indication) in the e-prescription system for each patient	Chapter IV forms are submitted once and does not track actual utilization, whereas IMA-AIM data is generated based on prescriptions	Blueteq forms are filled in by physicians but are only submitted once so they do not track actual utilization; SACT is driven by e-prescribing	Clinicians fill in information for *liste-en-sus* and specify the indication, which is linked to the PMSI	Each drug/indication has its own registry filled in by physicians, eventually allowing separate entry agreements (e.g., volume-price) for each combination component	Catalan data is based on e-prescriptions (automatic data input) and is checked against hospital invoices; physicians are expected to input data into Valtermed, but lack incentive	Physicians choose to enter data into the registries, but lack the incentive to	Health service provider notes down the indication code and sends a cost coverage request to the sick fund, who then approves the request for reimbursement as needed
*System integration*	Can be linked to other national datasets (not routine practice)	No official linkage between IMA-AIM and Chapter IV data	Fragmented since SACT does not fully capture Blueteq data and is not routinely linked	Integrated in the Health Data Hub – the Hub contains anonymized data in a secure platform; collects national health data (including PMSI) from existing data systems	Standalone	Catalan data collection is linked to prescription workflow and hospital invoicing system, and data can be exported to Valtermed	The same patient identifier is used across most data systems	Standalone
*Funding*	Nationally funded	Nationally funded	Nationally funded	Nationally funded	Industry	Catalan Health data system is funded by the Catalan Health Service, while Valtermed is nationally funded	Funded by regions	Industry
*Stakeholders involved*	Centrally managed by the national health authority	Data is aggregated from 7 sick funds and managed by AIM	Managed by NHS England	Centrally managed by the national health authority	Managed by the national health authority	Managed by the Catalan Health Service/national health authority	Academic societies	Third-party vendor aggregating data from different sick funds

**TABLE 4:** Data system rating by research team of current abilities to track utilization of combination therapies and potential to support combination-specific pricing.

​	Capability rating*	Tracking objective	Description
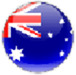	PBS database	Reimbursement claims data	• National claims data capable of tracking drugs by indication that differentiates between monotherapy and combination use • Although hospital drugs are not typically tracked, high-cost drugs (including oncology drugs like combination therapies) are included
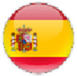	Catalan health registry	Medical records Reimbursement claims data	• The regional registry checks hospital invoicing data (reimbursement claims) against patients’ medical records, which tracks medicine use by indication • Reimbursement relies on invoicing so there is high incentive for physicians and pharmacists to enter invoices
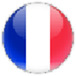	PMSI/SNDS database	Reimbursement claims data	• Claims data covers all *liste-en-sus* drugs, which includes innovative oncology drugs; the indication is recorded
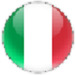	AIFA registries	Product registries	• Monitoring registries allow drug utilization tracking and can differentiate combination and monotherapy use, but note these registries are only for drugs that AIFA deems necessary for tracking, which are innovative or conditionally innovative drugs (majority of new cancer drugs fall in this category) • Though there are significant issues with data quality, coverage is high due to data collection being mandated by law and being linked to reimbursement
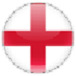	Blueteq and SACT dataset	Physician prescription requests record and Registry	• Oncology drug utilization (including combination therapies) can be tracked by indication through a cancer treatment register (SACT dataset) • The Blueteq high-cost drug management system requires physicians to complete a form for prescription, which can be used as a proxy for utilization
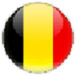	IMA-AIM dataset and Chapter IV physician prescription requests	Reimbursement claims data and physician prescription requests record	• Tracking hospital treatment usage is possible through claims data (IMA-AIM database) but not by indication • The ‘intention to treat’ requests by physicians (Chapter IV requests) can track indications and be used as a proxy for utilization
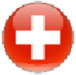	SmartMIP data	Reimbursement claims data	• Tracking utilization is enabled by a third-party vendor using claims data, but SmartMIP is not allowed to share data between different pharmaceutical companies (even if the companies have a combination therapy together) and there are significant issues with data quality • Lack of support from government authorities leads to inconsistent data collection, especially due to the federated health system that makes it difficult to drive coordination
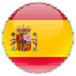	Valtermed	Disease registries	• A national registry (Valtermed) has been set up for treatment monitoring, but it only covers very high-cost drugs (e.g., rare diseases), and there are implementation issues
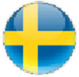	INCA, RCC	Various cancer registries	• There is no national database to track usage of hospital treatments by indication**,** but registries by scientific societies exist (funded by regions) • Though these registries are national in scope in theory, but coverage is poor in reality

#### 3.1.1 Data source

Countries have developed functional data systems within their own healthcare ecosystem based on a number of different types of data sources, including medical records, reimbursement (i.e., claims) data, product and disease registries, and prescription permission requests. In practice, different data sources can be used, or have the potential, to track the use of medicines in combination. For instance, the Italian Medicines Agency (AIFA) uses registries. However, these are product registries that track usage over time rather than patient registries that focus on tracking the patient’s condition (Xoxi et al., 2021). In contrast, Australia and France use claims data systems to capture the number of units prescribed and the indication for which the medicine was used, which can therefore track the utilization of medicines (in France, the *Programme de Medicalisation des Systemes d'Information*, PMSI, or hospital information system medicalization program, data is only available for products on the *liste-en-sus*) (Baker et al., 2018; OECD, 2019; Sturkenboom and Schink, 2021; Ministry of Solidarity and Health, 2024).

In Belgium and England, the Chapter IV “intention-to-treat” and Blueteq high-cost drug request forms, respectively, are based on systems where physicians request permission to prescribe certain medications and obtain reimbursement. Consequently, these requests can only be considered a proxy for actual utilization as it is unclear whether the requested medicines are prescribed, so data that reflects actual prescribing and dispensing such as claims data may be more appropriate. However, it is crucial to recognize that prescriptions do not necessarily translate into patient utilization. This may occur in cases where patients do not collect the prescribed medication or where the medication is not administered.

#### 3.1.2 Scope

The majority of data systems explored in this research include medicines across all therapeutic areas and aim to have national coverage. However, there are instances where the completeness of data regarding national coverage or disease range is not optimal. For example, Valtermed is a national registry in Spain that collects real-world clinical data to address uncertainty surrounding the long-term benefits of high-cost therapies, which technically has country-wide coverage (de Sanidad and Bienestar, 2019). However, such data systems are not optimal for tracking the use of medicines due to the selective coverage of specific diseases or drugs. In countries with strong regionality in their healthcare systems, there may also be issues where regions have their own siloed data systems that capture detailed information across diseases but lack national coverage, such as the health data system in Catalonia (Spain) and the highly decentralized healthcare system in Switzerland where data are fragmented at the sick fund level. To track the utilization of combination therapies, it is important that the data system should cover oncology comprehensively and should be a representative random sample of the use at the country level.

#### 3.1.3 Data granularity

**FIGURE 1:**
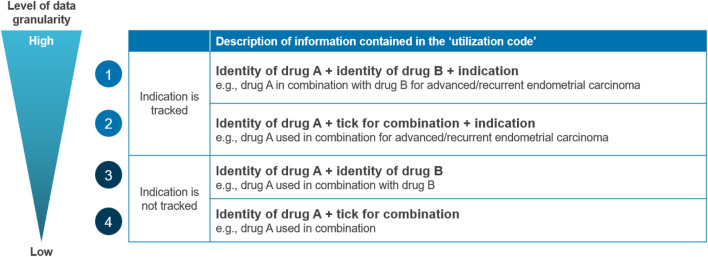
Data granularity cascade describing the potential level of detail captured on the utilization of a combination therapy. Source: Charles River Associates

The issue of data granularity is multi-faceted, as data systems capture different levels of detail (Figure 1), affecting their utility for tracking. When specifically considering oncology combination therapies, tracking utilization may be complicated by the fact that the constituents could be prescribed or used separately. Constituents may be used in sequence where one therapy is added to another after a period of time, which will require the use of one medicine initially as a “monotherapy” to still be captured as combination use. This issue also applies when one constituent is used for a shorter period than the other(s) (i.e., started in unison then one constituent is stopped before the other). Another complexity appears when one of the constituents is already on the market as a monotherapy (Drug A) and another component is an add-on that is only reimbursed as a combination therapy (Drug B). In these scenarios, there is a risk of underreporting the use of the combination if it is misreported as monotherapy use (especially if Drug A is used individually first). In some countries, such as France, there may also be cases where constituents are used in different settings of care (e.g., retail vs hospital pharmacies), which results in data on their use being captured in separate datasets and therefore requires robust data linkage capabilities. While France is able to link primary and secondary care data at the individual level, a significant limitation of the PMSI hospital data is that it only captures drugs on the *liste-en-sus*, which may complicate combination therapy tracking if only one constituent is on the list. However, it is important to note that these challenges may be less pertinent in countries where the tracking system can differentiate between monotherapy and combination indications.

The degree to which the data systems have sufficient granularity to allow tracking of combination usage versus monotherapy usage varies. Recording this distinction is possible in the PBS data system in Australia (reimbursement claims data) due to using different codes for logging each indication. For instance, a constituent has a different code when used as monotherapy versus use in combination therapy, and codes for the same product differ between indications. A similar mechanism is also applied in AIFA monitoring registries, with the caveat being this is only applicable to indications subject to monitoring, which are selected mainly because they are recognized as innovative, and this typically means that the constituent products are both novel products. It is important to note that reimbursement claims data systems do not always include the indication of a medicine prescribed and may need to be linked to other data sources to unlock this potential. A recent development was the Catalan Health data system consolidating a Patient and Treatment registry, merging prescription data with medical records that include clinical details and invoicing data, detailing the quantity of units used. This integration facilitates tracking of utilization by specific indications across all public hospitals in Catalonia (Roig Izquierdo et al., 2020; Pisana et al., 2022).

Detailed information obtained by monitoring usage based on indications permits comprehensive analyses, which are essential for distinguishing different uses. At the same time, determining use by indication may make the system too complex and increase the burden on data entry and analysis, considering that current medicines in disease areas beyond oncology can have an extensive list of indications. Moreover, collecting data on the indication leads to the concept of indication-specific pricing, which has many intrinsic challenges for implementation beyond the scope of this research (Mestre-Ferrandiz et al., 2018; Campillo-Artero et al., 2020).

Besides capturing the type of medicine and its indication, data systems also gather several important details essential for tracking the usage of medicines. These details include the number of unit(s) used, the start and end date of use, and rarely, the reason for the end of use. The collection of data on these variables varies between countries, and the format in which data is captured can also be different adding to the complexities. For example, the usage or sales unit can be recorded as the number of packs or vials, with or without dosage data.

#### 3.1.4 Data access and transparency

The details of personal information collected in data systems, such as electronic healthcare records (EHR), means that this information must be protected by strict data privacy laws. Consequently, access to data often differs considerably between patient-level and aggregated, de-identified, data. There are significant limitations across countries regarding the sharing of patient-level data due to privacy concerns and the potential usage for marketing purposes. Many countries only provide aggregated data from national data systems to the public and/or third parties. For example, in Australia, aggregated and de-identified data from PBS is publicly available, but third parties can purchase a random 10% sample of individual-level data (PBS10 dataset). This may be an issue for tracking usage of combination therapies where the usage may not be visible in this sample subset if the eligible patient volume is small. In France and England, access to PMSI and Systemic Anti-Cancer Therapy (SACT) data by third-party actors who are not government administrations is limited, and there is a relatively long and complex process to access the full dataset. Access can take up to 18 months in France for the full National Health Data System (SNDS) dataset, and can only be provided to third parties to “generate knowledge in the public interest” (OECD, 2019; Lestelle, 2023). Thus, despite having various rich data systems, the limited availability of detailed data to third parties can be problematic as this restricts the potential applications of the data to improve healthcare.

The purpose for which the data will be used is clearly an important determinant of how data are shared. For example, tracking utilization of products is important for rebate payments. As a result, Australia makes a monthly report available to manufacturers on the use of their drug(s) in different indications. In Italy, manufacturers are able to see the number of patients per indication by region. These data can then be applied to other purposes, such as combination-specific pricing. Beyond access, other measures, such as the irreversible pseudonymization of data and tracking of data management, which are applied in France, can reduce data privacy concerns.

#### 3.1.5 Data quality

Data quality is a key concern across data systems and robust auditing processes are necessary to maintain this. Routine auditing is a common practice in Australia, whereas Belgium and Italy do not audit data following their entry into the system, which leads to uncertainty regarding their accuracy. In Switzerland, the fragmented system involving multiple sick funds across multiple cantons further increases the risk of error. Beyond accuracy, it is important to consider the completeness of data entry. For instance, although the Swedish cancer registries, run by regional cancer centers, are theoretically supposed to have national coverage, this can be problematic as data entry by physicians is optional, leading to suboptimal coverage. To ensure completeness, certain elements of data systems are made mandatory in some cases. For instance, the input of the indication is required in Blueteq forms for reimbursement in England, which is further supported by using pre-set selections such as tailored regimen options based on indication to reduce the requirement for manual entry and risk of human error.

Another aspect that affects data quality is the timeliness of data. Most countries experience delays in the availability of up-to-date data. This delay is less of an issue when the availability of the latest data is aligned with local payment mechanisms (e.g., if data is updated once a year and reimbursement also happens annually), which does not occur in all countries. For instance, in Belgium, healthcare institutions are legally allowed to file invoices (reimbursement claims that could be used to track the use in combination) up to 2 years after the time of use, which does not coincide with the national reimbursement schedule.

#### 3.1.6 Data management and integration

Data input may also depend on healthcare professionals (HCPs) and can add a significant administrative burden, leading to low data completeness, which could lead to an underestimation of combination therapy utilization. Moreover, many countries have multiple IT systems and healthcare data system, which require significant integration efforts to obtain comprehensive and complete data sets. For example, gathering data for England’s SACT data system is made difficult by the different data infrastructure and prescribing systems across various hospitals, which burdens the technical team uploading data into SACT as they have to standardize the data to fit the SACT requirements.

Integrating data collection for tracking utilization into existing systems, such as electronic prescriptions or patient medical records, can reduce duplicative data entry efforts and burdens. This approach was the premise behind the Cancer Medicines Outcome Program (CMOP) programme in Scotland, where different datasets were pooled to unlock synergistic use cases and applications with minimal additional effort in data collection (Pisana et al., 2022). In another instance, the Catalan Health data system is a dedicated registry that currently requires specific input, but it is in the process of increasing automation by reducing the need for manual data transcription and achieving full linkage between medical records and invoicing data by 2025, and information systems are being re-designed to achieve this (Roig Izquierdo et al. 2020). Moreover, the linkage between submitting invoices and obtaining reimbursement means that physicians and pharmacists have a high incentive to enter data both accurately and in a timely manner, as demonstrated in the Catalan region.

#### 3.1.7 Funding

Across countries, most administrative data systems are integrated into the healthcare system and are therefore funded nationally. In contrast, AIFA monitoring registries and the SmartMIP data system in Switzerland are funded by the pharmaceutical industry, while the cancer registries in Sweden are maintained by the regional cancer centers which in turn are funded by the regions.

#### 3.1.8 Stakeholder involvement

Across the countries in scope, most data systems are managed by national authorities and/or dedicated data custodians. In some countries, the authority or data custodian is also a key policymaker. This can be beneficial because they are in a better position to fully utilize the data to inform their decisions and are, therefore, more aware of the data’s value. For example, AIFA’s management of the data systems ensures full utilization of data and control over sharing information between stakeholders (OECD, 2019). Because AIFA uses the data, there is an incentive for them to improve data collection and analysis, for instance by introducing increased automation. In contrast, where there is a lack of government support and lack of national prioritization of data collection, this can lead to inconsistent data collection since there are no consequences for poor data collection and entry by stakeholders. For example, there is a lack of government support to create a national data system for hospital medicines in Sweden, which means that only cancer registries (where data entry is not mandated) can be used to track oncology combination therapies used in hospitals. This is in contrast with the Swedish Prescribed Drug Register maintained by the Swedish National Board of Health and Welfare, which contains robust utilization data on medicines prescribed in pharmacies and has been extensively used in research as it is a rich data source that can be linked to other national registers (Wettermark et al., 2007; Wallerstedt et al., 2016; Xie et al., 2019; Socialstyrelsen, 2024). The utility of the data may also be undervalued as it is not fully utilized and considered in decision-making by policymakers, especially if there is a disconnect between the data custodian(s) and policymakers. This issue is demonstrated in Switzerland, where the health system involves multiple sick funds that collect data, but the lack of government involvement makes it difficult to drive coordination and leads to suboptimal data collection and utility in informing decisions.

In addition to national authorities, data custodians and policymakers, a key stakeholder group are the individuals responsible for data entry, which varies depending on the type of data system and the country, but often includes clinicians, pharmacists, hospital administrators and health insurance funds. Their commitment to data entry is crucial to the utility of the data system, so it is critical they share the benefits of data collection as this would incentivize their commitment.

### 3.2 Recommendations

After analysing data systems across selected countries, we explore recommendations to capture the minimum *data requirements* in terms of the coverage scope, level of data granularity and, finally, data protection in the context of stakeholder access. Following this, recommendations were developed to minimize the resources required to optimize the *tracking system infrastructure*, focusing on aspects related to the data source, data management and integration, data quality and stakeholder involvement. Consequently, the suggested recommendations aim to minimize both the amount of data and resources required across countries. Given each country’s different data systems and capabilities, further considerations should be made to account for diverse country contexts.

#### 3.2.1 Expert recommendations on minimum data requirements

##### 3.2.1.1 Scope of data

Recommendation: The establishment of a centralized tracking system is essential, initially focusing on data for oncology products, yet designed to be scalable to other disease areas. This foundational step is crucial for enabling data-driven and informed decisions for pricing and reimbursement.

Most pricing and reimbursement decisions are made on a national level; therefore, the tracking system for combination therapies should collect data at national level. This approach would also prevent introducing regional biases into data and ensure the conclusions drawn represent clinical practice within a country. Oncology should be considered a priority disease area for tracking as the pipeline for oncology combination therapies continues to expand, with 55 novel combinations expected to launch in Europe in the next 5 years, highlighting the urgency of combination-specific access solutions in oncology (Albrecht et al., 2020; IQVIA Institute for Human Data Science, 2023; Pistollato et al., 2023). As data capabilities mature in the medium to long term, coverage of tracking systems should be extended to other disease areas, using learnings from tracking oncology therapies as a “pilot program”.

##### 3.2.1.2 Data granularity

Recommendation: A tracking system should be able to monitor when a medicine is used as monotherapy or in combination, including the volume and/or the duration of the treatment as a combination through variables such as start date of use, dosage, and the number of units used.

Data systems should be able to differentiate the use of medicines, such as monotherapy versus combination. Ensuring that a medicine has a unique code for each indication it is reimbursed for, including different codes for use as monotherapy and in combination, could reduce misreporting for medicines that have multiple indications (especially if approved for both monotherapy and in combination). A robust tracking system must also be informative on utilization, as this would enable the implementation of combination-specific pricing and allow payers to implement rebates for products. Several variables should be collected to ensure that the volume of the use in combination is captured accurately: start date of use, dosage, and the number of units used (e.g., packs). This core set of variables should be standardized at the national level to ensure homogeneous data collection and easy analysis.

The need to track indications depends on the purpose of the tracking system. If the sole objective is tracking the utilization of combination therapies versus monotherapy, then tracking the indication is not necessary. However, if the system’s value is maximised beyond simply tracking combination use, such as to monitor the appropriate use of medicines, then the indication would be required.

##### 3.2.1.3 Data access

Recommendation: There is a need to create a data governance model to provide all relevant stakeholders secure access to certain agreed levels of data. At the same time, ensuring patient confidentiality remains paramount. To minimize the amount of confidential information handled, the focus should be on building upon administrative data, such as reimbursement claims, rather than detailed patient medical records to facilitate tracking while safeguarding patient privacy.

Enabling combination-specific pricing will require some level of access for the manufacturers of the combination therapy, given they are unable to distinguish between monotherapy and combination use with their sales data, especially when the other component is from a different company. Improving data accessibility is considered important, yet the imperative to protect patient confidentiality significantly reduces the willingness to share data from public data systems, particularly to third parties. To address such concerns, the tracking system must adhere to data privacy regulations, such as the General Data Protection Regulation (GDPR) and additional risk management strategies to guarantee personal data protection. Several European countries offer concrete models for enabling data access under strict privacy safeguards. For instance, France’s Health Data Hub aggregates data from multiple sources, including hospital discharge records and reimbursement claims, under a centralized platform where all personal identifiers are replaced with pseudonyms generated through a two-step encryption process. Access is granted only within a secure cloud environment to approved users, with oversight by the national data protection authority (CNIL) (European Health Information Portal, 2022a). In Sweden, data access is facilitated through the National Quality Registries and the Statistics Sweden MONA system, which allows researchers to use pseudonymized datasets within highly secure, virtual environments. Personal identity numbers (PINs) are replaced with non-identifiable codes, and analyses are conducted in environments that prevent export of raw data, aligning with GDPR-compliant “safe settings” principles (European Health Information Portal, 2022b; Statistics Sweden, 2023). Italy provides another example through the Italian Medicines Agency (AIFA) registries and its broader national data governance policies. Data for secondary use, such as for research or health planning, must be de-identified at the source, with access strictly controlled through formal agreements, ethics approval, and in many cases, review by the national Data Protection Authority (Hansen et al., 2021). Italy mandates that only the minimum necessary data be shared, and only after pseudonymization or anonymization procedures (Van Quathem and Somaini, 2023). The European Commission stipulates that access to EHDS health data by researchers, innovators, public institutions or industry should only be granted under strict conditions, such as if the data requested is used for a specific purpose in a secure environment without disclosing individual identities (Horgan et al., 2022). As such, administrative data including reimbursement claims information, should be favoured over detailed patient medical records as a starting source of data for tracking that can be further optimized. National authorities should govern the tracking system and the level of access given to third parties, including pharmaceutical companies. Whilst patient-level data may not be appropriate to share with third parties, aggregate data may be insufficient for companies to engage in pricing and reimbursement negotiations. Consequently, access to an intermediate level of detail could be designed through engagements between data authorities and national pharmaceutical industry associations (and involving other relevant stakeholders). For example, while third parties should not be able to access patient-level data routinely, *ad hoc* queries of aggregated data, such as by indication, could be allowed. Contracts should be signed to ensure that data are used appropriately.

There may also be value in appointing an independent, trusted third party within a country to oversee the tracking system and compile fully anonymized data for controlled access by pharmaceutical companies. These could be academic institutions or publicly funded non-profit organizations, who would ensure that the analysis, aggregation and sharing of data is conducted in an unbiased manner (Michelsen et al., 2020).

#### 3.2.2 Expert recommendations to minimize resources required to optimize tracking infrastructure

##### 3.2.2.1 Data source

Recommendation: Healthcare systems should leverage existing data systems as a foundation and optimize them to enable tracking the usage of oncology combination therapies.

Healthcare systems should explore synergies between existing data systems and optimize data linkage to avoid duplication of data sources and administrative efforts. Integrating the tracked use of combination therapies into existing data collection practices and leveraging existing administrative data systems should be considered viable for achieving low-burden data collection. In many countries, reimbursement claims data are generally considered a good starting point for tracking the utilization of medicines and could be supplemented with other data sources if necessary, including hospital data. For instance, considering the potential for significant delays in the availability of updated reimbursement claims data, prescription authorization data could be used as a temporary proxy for utilization.

Given the added complexities in tracking the utilization of combination therapies, existing data systems might not be entirely prepared to address these challenges and could require specific enhancements. A crucial initial action would be to establish a consensus on the reporting of combination usage. Achieving agreement on recording combination therapies would lead to more precise data collection and minimize confusion in data analysis.

##### 3.2.2.2 System integration and data management

Recommendation: In addition to using existing databases, it is important to focus on automation and interoperability across data systems as a next step, to ensure efficient, low-burden data collection on combination therapy utilization.

Effective data collection relies on substantial human and financial resources (OECD, 2019; Pisana et al., 2022). Rather than starting from scratch, most countries may just need infrastructure upgrades or new organizational circuits to enable new functions and reduce current barriers to establish a robust data collection infrastructure across sectors. For example, different data systems containing patient and reimbursement information should ideally operate in an automated manner as well as be linkable and interoperable using common data formats. Efforts to standardize heterogeneous IT systems and data infrastructures between hospitals and regions will be needed to increase interoperability and easy data aggregation. The Observational Medical Outcomes Partnership Common Data Model developed by the Observational Health Data Sciences and Informatics (OHDSI) is an example of an initiative to standardize healthcare data for systematic analyses (Biedermann et al., 2021).

##### 3.2.2.3 Data quality

Recommendation: Adoption of digital and integrated prescribing systems will help ensure adherence to standards and enable timely, accurate data input.

Data quality and completeness are major limiting factors in tracking ability. One driver for poor data quality is the need for manual data entry, so introducing more automation could mitigate this barrier (Pisana et al., 2022). For instance, implementing predefined treatment lists tailored to specific patient conditions (closed prescription protocols), and utilizing existing oncology algorithms can streamline the process. These lists should be regularly updated to reflect new therapies and indications in updated treatment guidelines. At the same time, HCPs must also be encouraged to enter data correctly despite the perceived burden.

To promote completeness of data, it is necessary to incentivize robust data entry through a multi-faceted approach. National health authorities may wish to enforce mandated data entry by making certain variables (e.g., the indication) mandatory for prescription and/or reimbursement. To ensure accountability, routine audits with meaningful consequences for incomplete or inaccurate data entry should be implemented. Audit results could be reported as statistics to compare performance between regions and be used to allocate a percentage of rebates as a reward for data entry. Moreover, giving individuals entering data opportunities to use and access the data for research purposes may incentivize efficient and complete data collection. When considering the timeliness of data, availability of up-to-date data should be aligned with local payment mechanisms, which would differ between countries. In certain cases, the timeliness of data is constrained by legislation (e.g., allowing reimbursement claims to be submitted up to 2 years after), in which case an ‘as soon as possible’ approach is satisfactory.

It is important to note that any measures taken to improve data quality and completeness should not increase the burden on individuals entering the data, as this could dissuade data entry (Roig Izquierdo et al., 2020; Pisana et al., 2022). For instance, mandated data entry with strict controls and measures could increase administrative burden. To achieve a balance between efficiency and the burden of data entry, there is a need to engage in a collaborative approach to establish a digital automated system with routine audits to maintain data integrity. Consequently, a compromise needs to be reached through multi-stakeholder discussions and potentially optimized through experiences with pilot systems, and through this gauge the appetite for full implementation.

##### 3.2.2.4 Stakeholder involvement and funding

Recommendation: The tracking system for combination therapy usage should be governed by national authorities, ensuring authoritative oversight and consistency, while its design should actively involve various stakeholders including HCPs, industry representatives, IT experts, and patient groups, to create a system which is useful to as many stakeholders as possible. The value of tracking should be tailored to each stakeholder group and communicated clearly to encourage support and engagement. Further discussions will be required to determine the best source of funding to support tracking, considering the range of stakeholders involved.

Involving multiple stakeholders and establishing an overarching governance structure are essential to ensure that the system addresses diverse needs effectively and maximizes its utility and value. Stakeholders should include national health authorities, health data authorities, pharmaceutical companies/industry association(s), IT specialists, payers, health insurance funds and patients (or patient representatives). The involvement of the national health authority is key, as a system that is mandated and endorsed by these bodies will promote data collection.

National authorities should advocate the value of tracking across stakeholders to promote collaboration and ensure cohesive efforts. However, each stakeholder group’s distinct interests and incentives could hinder cooperation. Several authors have advocated for conducting a comprehensive analysis of the interests of the different stakeholders, which could identify potential conflicts of interest and affiliations that may hinder the implementation and execution of a tracking system for combination therapy usage (Pisana et al., 2022). It was also identified that a barrier to collaboration is the lack of trust between manufacturers, payers and HCPs, which could, for example, lead to disagreement on data access (Pisana et al., 2022). Thus, all stakeholders should be encouraged to respect any agreements or compromises reached and should act transparently and be open about their goals. In Italy, the AIFA monitoring registries are a multi-stakeholder collaboration between industry and AIFA, and despite having clashing preferences on aspects such as data sharing, a balance in objectives has been achieved for over a decade (Xoxi et al., 2021).

Based on this, a comprehensive list of key messages for tracking can be developed and potentially tailored to suit individual needs when communicating with different stakeholders. For example, while the utility of data for pricing and access might be attractive for payers, this can be less important for physicians and so would not be a major driver for data entry. In contrast, tailoring the message to emphasize the increasing concerns with regards to the availability of resources to fund new therapies, and the need to ensure resources are used as efficiently as possible to benefit all patients, may resonate more with healthcare providers. A better initial motivator for data entry may be using data to audit to what extent clinician prescription practices align with recommended, evidence-based guidelines and alongside this, help assess the impact of new therapies on patient care in practice (e.g., benefits from increasing the use of combination therapies) and allowing experience sharing between centers in case of heterogeneity (Roig Izquierdo et al., 2020). To better illustrate the value to stakeholders, pilot projects could be run, which present a learning opportunity to understand any significant challenges that may hinder the value for different stakeholders. It is important to note, however, that usage of data beyond tracking the utilization of oncology combination therapies will require additional features, such as recording the indication, which is beyond the scope of this study. Thus, the wide-ranging benefits of improved data collection could be explored with stakeholders while keeping in mind the potential need for additional capabilities.

If stakeholders are fully aligned on the value of the tracking system and that it meets the needs of as many stakeholders as possible, funding is more likely to follow. Funding is important to address limitations in the ability of current data systems. This potentially includes expanding the scope of current data systems within countries to cover both hospital and ambulatory care, improve data quality through routine auditing, develop data protection measures without compromising access and enable linkages between different systems.

## 4 Discussion

The main objectives of this study are to investigate the ability of current data systems to track oncology medicine utilization across several European countries and Australia, and use these learnings to construct recommendations that will assist policymakers in establishing a robust tracking system for combination therapies. Across countries in scope, the findings underscore the significance of existing data sources, particularly administrative data, as a valuable form of real-world data that offers insights into medicine usage that can be used to guide policy decisions (Clopes et al., 2017; Eriksson et al., 2018). Our findings show that there is substantial variation in the availability and capabilities of the data systems routinely collecting medicine utilization data across countries. Differences are in part driven by the reason for data collection, including reimbursement claims data, medical records and product registries, which leads to individual unique data system profiles. Some can be considered best practice examples across the parameters assessed, such as the PBS data system in Australia, in terms of data granularity, access and quality, while other countries are lacking when it comes to tracking the use of hospital oncology medicines.

Beyond country-specific nuances, overarching themes that emerged include concerns regarding fragmented data sources, data quality, standardisation variability, and challenges with system interoperability. The findings illustrate broader patterns in data capabilities that highlight general needs across European countries, drawing attention to the need for infrastructural improvements and increased awareness and advocacy efforts. Most countries already have data systems that can function as the backbone for additions that can then enable tracking utilization but, nonetheless, will require optimisation for use and proper implementation, potentially through pilot programs. Identified issues and concerns are likely to be seen across countries.

Increasing access to medicines is a key component of the EU Pharmaceutical Strategy, showcasing the EU-wide commitment to this issue (European Commission, 2020). Creating a blueprint for a robust tracking system in oncology can set the pathway to expand its application to other therapeutic areas and cover a broader spectrum of treatments, particularly high-cost medicines, given they face the most budget impact concerns and access challenges (EFPIA, 2020). Tracking medicine utilization can promote value-based healthcare and improve patient access by enabling novel payment models, including combination-specific pricing. In addition, it can reduce unintended heterogeneity in the uptake of medicines and ensure appropriate use and promote better care quality. This is important for priority disease areas such as cancer with growing morbidity, mortality and costs. For example, the AIFA monitoring registries in Italy primarily serve drugs whose therapeutic indications have been recognized as innovative, and in other cases can be used as tools during price negotiation (including non-innovative drugs/indications) and finally, to ensure prescription appropriateness to avoid off-label use (Xoxi et al., 2021; Xoxi et al., 2022). Similarly, the systems in Catalonia can track the usage of medicines across sectors, the adherence to guidelines, and outcomes to assist with managed entry agreements and other administrative schemes (Björkhem-Bergman et al., 2013; Clopes et al., 2017).

Considering the different components of a data tracking system and the realistic needs of the different stakeholders in healthcare, we identified a set of recommendations on how to enhance data collection infrastructures and build a future-proof tracking system that can deliver benefits to oncology patients first, then subsequently be expanded to other therapeutic areas and achieve additional objectives. To design a system for tracking the utilization of combination therapies, it is crucial to balance what components an ideal system could have, what is feasible in reality, and what is considered valuable to all key stakeholders. Thus, a compromise needs to be struck between the level of detail captured in the system, the administrative burden it poses, the quality of data collected, and the utility of this data (Pisana et al., 2022). In the ideal scenario, the data collected would be highly granular and of good quality while posing minimum disruption to existing workflows and little additional resource use. In reality, although collecting more granular data and implementing processes to ensure quality will increase the utility of data, the resulting increase in administrative burden could dissuade stakeholders. Whilst no one-size-fits-all solution exists, there are recommendations on how to optimize data granularity, quality and administrative ease for an effective and sustainable system.

The recommendations outlined in this research can be extrapolated in a disease-agnostic manner to inform policy decisions that improve healthcare data infrastructures more generally. At the core, it is crucial for policymakers to explore the benefits of enhancing and expanding data infrastructures synthesized from various country experiences. The broader implication of adopting these structures is the prospect of ensuring more rapid patient access to innovative treatments across therapy areas and improving patient outcomes, moving towards value-based healthcare that optimises the use of healthcare resources through data-driven decision-making. Ultimately, this will benefit all key stakeholders going forward.

### 4.1 Strengths and limitations

We believe a strength of this study was that it created new insights in an area which hasn’t been researched sufficiently but seems to be a basic requirement to improve access to medicines. The primary research was conducted across a wide range of countries and involved senior multi-disciplinary experts in each country with extensive expertise in health data systems and oncology. The open-ended questions in individual interviews and a semi-guided discussion involving all experts allowed the capture of country-specific nuances as well as general learnings that can be applied internationally. This allowed for an in-depth review of relevant data systems in each country and yielded detailed analyses from which the recommendations were derived. All topics of discussion achieved a strong consensus amongst participating experts, ensuring the findings are applicable to the countries in scope and likely beyond these.

Nevertheless, we are aware that this study has several limitations. The premise of the research is based on improving access to combination therapies through solutions requiring improved data systems. While the lack of data on utilization is foundational in driving pricing and access challenges for combination therapies, it is important to acknowledge there are many other challenges that impact these therapies, which were not the focus of this study and were therefore not addressed. Although the range of countries selected allowed for exhaustive exploration of both best practices and limitations, the scope could have been expanded to include additional regions and countries that can offer other perspectives. Furthermore, the number of experts interviewed was somewhat limited due to the strict selection criteria on their knowledge of data systems and local country data systems.

We also opted for a targeted literature review in response to our highly specific research question, choosing to primarily source information from official websites. This approach allowed us to access the most current data directly relevant to our topic, ensuring accuracy and timeliness crucial for the rapidly evolving nature of the issue at stake. However, we acknowledge that this focus may have limited the breadth of our review, potentially omitting varied perspectives typically found in academic journals. While this strategy sacrificed some comprehensiveness, it was necessary to achieve the depth and specificity required to address our research objectives effectively.

## 5 Conclusion

This research was conducted in the context of combination therapies in oncology. There is a consensus that combination-specific pricing can accelerate and broaden patient access. However, this requires a tracking of the use of therapies. Improving the tracking of combination therapies can ameliorate the unique pricing and reimbursement challenges impacting these therapies. At the same time, enhancing the tracking of medicine utilization beyond combination therapies is foundational to improving the quality of patient care, evaluating the alignment of medical standards with clinical guidelines, and controlling the allocation of resources. To support the robustness of data collection and their application, buy-in from a range of stakeholders other than policymakers is required. Overall, it is essential to acknowledge the divergence in the perceived value of tracking across countries and stakeholders, given their unique priorities and dynamics.

## Data Availability

The original contributions presented in the study are included in the article/Supplementary Material, further inquiries can be directed to the corresponding author.
