# Correction: The unexploited potential of data systems tracking medicines utilization: an opportunity to improve access to oncology combination therapies

**DOI:** 10.3389/fphar.2025.1693232

**Published:** 2025-09-05

**Authors:** Steven Simoens, David Dodwell, Michael Hartevelt, Peter Lindgren, Michele Pistollato, Lisa G. Pont, Caridad Pontes, Alexander Roediger, Antun Sablek, Eric Van Ganse, Qinyi Wang, Chris Wenger, Tim Wilsdon, Entela Xoxi, Brian Godman

**Affiliations:** ^1^ KU Leuven Department of Pharmaceutical and Pharmacological Sciences, Leuven, Belgium; ^2^ Oxford Population Health, University of Oxford, Oxford, United Kingdom; ^3^ Global Oncology Policy, Merck, Rahway, NJ, United States; ^4^ Swedish Institute for Health Economics, Lund, Sweden; ^5^ Health Economics, Karolinska Institute, Solna, Sweden; ^6^ Charles River Associates, London, United Kingdom; ^7^ Graduate School of Health, University of Technology Sydney, Sydney, NSW, Australia; ^8^ Department of Pharmacology, Therapeutics and Toxicology, Autonomous University of Barcelona, Barcelona, Spain; ^9^ Charles River Associates, Brussels, Belgium; ^10^ Pharmacoepidemiology Unit, Claude Bernard University Lyon 1, Lyon, France; ^11^ Charles River Associates, Cambridge, United Kingdom; ^12^ Aquantic AG, Zeiningen, Switzerland; ^13^ Postgraduate School of Health Economics and Management (ALTEMS), Catholic University of the Sacred Heart, Rome, Italy; ^14^ Strathclyde Institute of Pharmacy and Biomedical Sciences, University of Strathclyde, Glasgow, United Kingdom; ^15^ Department of Public Health Pharmacy and Management, School of Pharmacy, Sefako Makgatho Health Sciences University, Pretoria, South Africa

**Keywords:** oncology combination therapies, individual patient-level healthcare data, utilization tracking, pricing and reimbursement, pharmaceutical policy, Europe, Australia

Author Entela Xoxi was erroneously assigned to **affiliation** Strathclyde Institute of Pharmacy and Biomedical Sciences, University of Strathclyde, Glasgow, United Kingdom. This affiliation has now been removed for author Entela Xoxi.

Author Lisa G. Pont was erroneously spelled as Lisa Pont.

The original article has been updated.

