# Supplementary material for: The unexploited potential of data systems tracking medicines utilization: an opportunity to improve access to oncology combination therapies
Source: Front Pharmacol. 2025 Aug 20;16:1532022. doi: 10.3389/fphar.2025.1532022 (PMC12406276; doi:10.3389/fphar.2025.1532022)
Supplement: Supplementary file 1 [file Supplementaryfile1.docx]

Supplementary Material

# Specific Data System Landscapes by Country

## Australia

Table 1. Pharmaceutical Benefits Scheme (PBS) Data System

| Framework component | Details |
| --- | --- |
| Data source | - The PBS database comprises of data routinely collected on the dispensing of medicines listed on the PBS for the whole population – the database was established for administrative and payment purposes but has been used for routine monitoring, surveillance, and research for many years^^[[1]](#endnote-2)^^ - PBS claims data are an administrative by-product of the Department of Human Services (DHS) administration of the subsidized prescription payment system^^[[2]](#endnote-3)^^ - It facilitates rebate payments from industry – a rebate is paid for each individual indication^^[[3]](#endnote-4)^^ - The Department of Health issues separate rebate invoices, referencing separate rebate amounts, for each indication as per the listing agreement made following the positive reimbursement assessment^^[[4]](#endnote-5)^^ - Collected data can potentially be considered to inform price-setting or reimbursement decision^^[[5]](#endnote-6)^^ |
| Scope | - The PBS database is a standalone database that covers the entire Australian population^^[[6]](#endnote-7)^,^[[7]](#endnote-8)^^ - The PBS database does not generally include hospital drugs, but oncology drugs are captured, especially high-cost medicines and combinations^^[[8]](#endnote-9)^^   - Chemotherapy agents are considered low cost and may not be tracked as a result^^[[9]](#endnote-10)^^   - To be listed on the PBS, the drug must be assessed and recommended by the Pharmaceutical Benefits Advisory Committee^^[[10]](#endnote-11)^^ |
| Data granularity / completeness | - The ‘authority code’ is usually given to medicines that are high-cost and/or have safety concerns, and these medicines are tracked in the PBS database^^[[11]](#endnote-12)^^   - Each approved indication is assigned a unique authority code, and is quoted by the prescriber on the prescription - The PBS item code and streamlined authority code include the approved indication – it is possible to differentiate use in monotherapy and combination therapy^^[[12]](#endnote-13)^,^[[13]](#endnote-14)^^   - PBS item code: PBS defined codes that provide medicine details (e.g., generic name, approved indication, etc.)   - Authority codes are used for high-cost medicines or medicines with uncertain safety profiles^^[[14]](#endnote-15)^^ - The date of prescription and date of supply/dispensing are provided (sometimes only one is provided, sometimes both), as well as the number of prescriptions dispensed (including original and repeat prescriptions)^^[[15]](#endnote-16)^^ - Although the number of units is included, the dose and duration of use is not recorded in the PBS dataset, but national protocols are clear for oncology so it could be assumed that use is standardized^^[[16]](#endnote-17)^^ |
| Data access / transparency | - Pharmaceutical companies get a monthly report on the use of their drug(s) in different indications and can purchase data – a separate rebate is then paid for each different indication^^[[17]](#endnote-18)^,^[[18]](#endnote-19)^^   - Companies can obtain reports on other companies’ products, but they will not contain confidential information like rebates^^[[19]](#endnote-20)^^ - Industry can access PBS data – aggregated/de-identified data is publicly available^^[[20]](#endnote-21)^,^[[21]](#endnote-22)^^ - If industry wishes to purchase individual-level data, they can purchase a 10% sample – however, if combination use is too low, it will not be visible in the 10% sample^^[[22]](#endnote-23)^^ |
| Data quality | - The database is highly accurate due to its administrative purpose and real-time, automated collection of claims data by pharmacies^^[[23]](#endnote-24)^^ - Data quality is considered robust due to the electronic prescription system – this reduces the need for manual entries, so the data is quite accurate^^[[24]](#endnote-25)^^ - Authority codes are used to infer patient diagnosis/indication; this relies on the doctor or pharmacist selecting the correct code^^[[25]](#endnote-26)^^ - The PBAC conducts audits – they check if item numbers are used for the wrong indication, or if the volume is too high for one drug, etc.^^[[26]](#endnote-27)^^ - Data can be obtained monthly to pay rebates^^[[27]](#endnote-28)^^   - But data is usually delayed by 6 weeks, while competitor data is available with a 6-8 week delay^^[[28]](#endnote-29)^,^[[29]](#endnote-30)^^ |
| Data management | - Physicians need to use the item code in electronic prescribing program to get reimbursement^^[[30]](#endnote-31)^^   - Each approved indication is assigned a unique authority code, and is quoted by the prescriber on the prescription - The e-prescription system allows clinicians to pick a drug, and then a list of indications pops up for clinicians to select^^[[31]](#endnote-32)^^ - There is a monthly automated system that provides an invoice to the market access holder by indication^^[[32]](#endnote-33)^^ - The Medicaid number is used as the unique identifier number for patients^^[[33]](#endnote-34)^^ |
| System integration & funding | - The PBS data tracking system is integrated in the healthcare system – data is recorded when physicians enter a prescription for reimbursement^^[[34]](#endnote-35)^^ - Funded at a national level and is provided by the same funding mechanisms to finance healthcare^^[[35]](#endnote-36)^^ |
| Stakeholder involvement | - The PBS database is fully under public health authorities – the data custodian of the PBS claims database is the Australian Department of Health^^[[36]](#endnote-37)^^ |
| General comments^[[37]](#endnote-38)^ | - Australia has already implemented IBP (different price is negotiated for different indications) - Since price, rebates and funding are set at the federal level, tracking is conducted at the national level - Instead of establishing a tracking system, countries should think about using existing data and datasets that can be used for tracking to ensure more efficient use of data - There may be higher distrust for the data if industry funds tracking - Drugs in combination are not always used in combination – sometimes, one of the drugs will be stopped earlier than the other, but a mechanism will need to be introduced to ensure that it is still captured that the drug is still being used as part of a combination - The number of packs will be needed if pricing is determined by the number of vials - Electronic prescribing should make it easy to build in tracking capabilities |

## Belgium

Table 2. Inter-Mutual Agency (IMA-AIM) and Chapter IV ‘Intention to Treat’ Data Systems

| Framework component | Details |
| --- | --- |
| Data source | - Physicians must request for reimbursement for a specific indication for innovative drugs which are all reimbursed under Chapter IV^^[[38]](#endnote-39)^^   - When prescribing a product from Chapter IV, the physician fills out a form to request approval of the advising physician of the health insurance fund – the form is filled out electronically through an online web application CIVARS (includes indication)   - If an approval is granted, the approval with relevant information is stored in an approval repository - The IMA-AIM database contains routinely collected data on prescribed or dispensed medicines based on claims data^^[[39]](#endnote-40)^^   - The data is collected by 7 Belgian sick funds and aggregated to a national level database, then IMA makes them available for research purposes – the data is patient-level and is anonymized^^[[40]](#endnote-41)^,^[[41]](#endnote-42)^^   - Unlike prescription authorization data, IMA-AIM claims data does not track the indication^^[[42]](#endnote-43)^^ |
| Scope | - IMA-AIM data captures all reimbursed prescription drugs, including innovative drugs under Chapter IV reimbursement authorization^^[[43]](#endnote-44)^^ - All innovative medicines are under Chapter IV^^[[44]](#endnote-45)^^   - For combination therapies, automatic inclusion in Chapter IV is dependent on both drugs being on patent; if one of the components is a generic, then the combination might not be included (unless the combination is still deemed to have significant financial burden)^^[[45]](#endnote-46)^^ - Hospital drugs under Chapter IV can be tracked through the Chapter IV reimbursement authorization system (intention to treat) as well as claims data/billing data^^[[46]](#endnote-47)^^ |
| Data granularity / completeness | - The IMA-AIM database contains routinely collected data on prescribed or dispensed medicines based on claims data, but does not include the indication^^[[47]](#endnote-48)^^ - Chapter IV data does include the indication, but is not routinely linked to IMA-AIM data^^[[48]](#endnote-49)^^ - Claims data itself cannot track indications – but could potentially be linked to the Chapter IV authorization data, which does include the indication^^[[49]](#endnote-50)^^ - Data is organized by patient, and the patient is identified by a national identifier^^[[50]](#endnote-51)^^ - IMA-AIM data captures the number of packs, which can then theoretically be used to identify whether the product was used appropriately^^[[51]](#endnote-52)^^ |
| Data access / transparency | - Pharmaceutical companies IMA-AIM claims/billing data, but not Chapter IV data (Chapter IV data is not available to third parties)^^[[52]](#endnote-53)^^   - It should be noted that Chapter IV data has never been used for other purposes, even research^^[[53]](#endnote-54)^^ - With the IMA firewall, once a company has a managed entry agreement on a product, they can request data on that product and nothing else^^[[54]](#endnote-55)^^   - Access to IMA data is a complex process - it takes a lot of time to set up an agreement with IMA-AIM (e.g., study protocol) which could take 6 months+, and permission from the privacy commission is also needed^^[[55]](#endnote-56)^^ |
| Data quality | - Claims data is based on different platforms from different sick funds, so the quality is not ideal as there is missing data, double entries, etc.^^[[56]](#endnote-57)^,^[[57]](#endnote-58)^^ - Quality of Chapter IV requests are not audited^^[[58]](#endnote-59)^^   - A drug with multiple indications and different combinations will have different codes, and there are checkboxes that specify the indication that the physicians can tick, but the selection of the indication is not really audited   - Since the indication selection is at the discretion of doctors, and it is not really checked if the indication is correct, the data quality may be affected - IMA-AIM data is subject to huge delays, since invoicing from hospitals can be done after 2 years^^[[59]](#endnote-60)^^   - On average, there is a 7 to 9-month delay, so data is not a live snapshot of utilization - Due to the fact that Chapter IV data is updated live, it could potentially be used as a proxy for utilization until the IMA-AIM claims data is updated^^[[60]](#endnote-61)^^ |
| Data management | - All indications for a drug like Keytruda are under 1 chapter in Chapter IV, but there are checkboxes for doctors to select the correct indication^^[[61]](#endnote-62)^^ - However, for Chapter IV requests, physicians usually only request the medication once (not for prescription renewals), so actual utilization is not captured^^[[62]](#endnote-63)^^ |
| System integration & funding | - No official linkage between IMA-AIM and Chapter IV pre-authorization data to capture indications^^[[63]](#endnote-64)^^   - It would be ideal if the claims data can be linked to the authorization data so that the actual utilization can be tracked - Both the Chapter IV authorization data and the IMA-AIM claims data are integrated in the healthcare system^^[[64]](#endnote-65)^^ - Nationally funded |
| Stakeholder involvement | - Data is aggregated from 7 sick funds and managed by IMA-AIM^^[[65]](#endnote-66)^^   - IMA-AIM claims/billing data are held by sick funds, and IMA-AIM can retrieve this data - Chapter IV data is managed by the National Institute for Health and Disability Insurance (INAMI)^^[[66]](#endnote-67)^^ - Belgium has introduced a health data authority to ensure linkage between databases^^[[67]](#endnote-68)^^   - While Belgium has multiple sick funds, there are federated bodies that are in charge of aggregating data across these funds, which allows national/centralized data collection^^[[68]](#endnote-69)^^ |
| General comments^[[69]](#endnote-70)^ | - Belgium has introduced a health data authority to ensure linkage between databases - There is general suspicion for pharmaceutical companies to be granted access to databases like IMA-AIM - It may be beneficial to set up a trusted third party that can access highly sensitive data from databases like IMA-AIM and Chapter IV in order to track, such as the Belgian Healthcare Knowledge Center - While Belgium has multiple sick funds, there are federated bodies that are in charge of aggregating data across these funds, which allows national/centralized data collection (unlike Switzerland where there is no national body and data is therefore extremely siloed) |

## England

Table 3. Blueteq Prescription Authorization Data and the Systemic Anti-Cancer Therapy (SACT) Data Systems

| Framework component | Details on Blueteq | Details on SACT |
| --- | --- | --- |
| Data source | - Blueteq is a prescription authorization system (clinical decision support tool) whereby physicians have to fill in a form to obtain funding for high-cost drugs^^[[70]](#endnote-71)^,^[[71]](#endnote-72)^^ - It enables funding bodies to monitor the use of expensive treatments and only reimburse if used appropriately^^[[72]](#endnote-73)^^ | - The SACT dataset collects information on the use of systemic anti-cancer therapies across all NHS England trusts to support optimal use, understand resources required for service provision and support commissioning decisions and understand the cancer patient pathway through linkage to other data sources^[[73]](#endnote-74)^ - It is a mandatory collection that enables an understanding of treatment patterns and outcomes^[[74]](#endnote-75)^ |
| Scope | - Most high-cost oncology drugs require Blueteq pre-authorization, not just those under CDF^^[[75]](#endnote-76)^^ - Due to the commercial sensitivity of data captured by Blueteq for drugs under the CDF, data in Blueteq for these products might not be included in the SACT^^[[76]](#endnote-77)^^ | - All chemotherapy drugs and most novel oncology therapies are captured^[[77]](#endnote-78)^ - Older drugs and supportive drugs (e.g., endocrine therapy, dexamethasone) are not prescribed through e-prescriptions, and so are not routinely included^[[78]](#endnote-79)^ - Drugs in the CDF may not be captured in the SACT due to the commercial sensitivity surrounding the CDF^[[79]](#endnote-80)^   - This may be an issue for combination drugs if one component is under the CDF |
| Data granularity / completeness | - Includes the indication, line of therapy and whether a drug is used as a monotherapy or combination, etc.^^[[80]](#endnote-81)^^ - Invoice to NHS England and Blueteq both contain a patient’s unique identifier, so data is at the patient-level^^[[81]](#endnote-82)^^ | - In theory, can recognize use in combination vs. monotherapy by indication^[[82]](#endnote-83)^   - SACT tracks the regimen (e.g., combination), but also tracks use of the components in the regimen, but whether it is used in combination cannot be easily queried (need algorithms to enable this)   - However, the stage and morphology of cancer is not well-completed which may make it difficult to identify the indication   - SACT data can be linked with the cancer registry that can provide other information (including indication) - Includes: unique patient identifier, treatment, indication, start date of use, end date of use, the reason for end of use, dosage^[[83]](#endnote-84)^   - The number of packs is not recorded   - Date of last treatment is recorded but the reason for end of use is not well completed - Patient-level data is pseudo-anonymized with a confidential identifier |
| Data access / transparency^[[84]](#endnote-85)^ | - Not accessible to pharmaceutical companies | - Pharmaceutical companies can access SACT data at cost, and must go through the governance/ethics process – thus companies prefer to use intermediaries like IQVIA - There is concern by patient bodies and charities about confidentiality and misuse of data for commercial purposes |
| Data quality^[[85]](#endnote-86)^ | - Data quality is relatively high as physicians are required to fill the form in to obtain reimbursement, and it is used for rebates | - The e-prescription system uses a preset algorithm, which includes regimen options as defined by the hospital - NHS introduced levers to improve data collection – e.g., if performance status is not entered, then no prescription allowed (since it has been made compulsory, performance status is now completed 80-90% of the time) - It is also very accurate in terms of discontinuation of treatments - Data is updated every 2 months, but there can be considerable delays between the time the drug is prescribed and when the data are captured (up to 12- 18 months) because data is quality assured and checked; trusts can also fall behind on uploading data   - Prescriptions today would be available in the Christmas 2024 dataset |
| Data management^[[86]](#endnote-87)^ | - The Blueteq form has criteria for prescription that physicians must fulfil to receive approval - Blueteq data captures the indication, which allows pharmaceutical companies to pay specific rebates   - However, Blueteq data is not utilization data – some patients do not start treatment despite completion of a Blueteq form (NHS England estimates that ~95% of patients do commence treatment) - HCPs will fill in a Blueteq form at the point of initiation but not at each subsequent infusion   - It therefore does not track utilization (patient may not comply to pre-defined treatment course) | - SACT derives data from the prescribing system for oncology drugs within a hospital   - However, a challenge in aggregating this data into a national database is the fact that there are ~5-6 different prescribing systems in England (some are unique to one hospital)   - Uploading data to SACT is difficult when there are a variety of different prescribing systems across individual hospitals - The pharmacy team is responsible for uploading data into the hospitals’ system, which is linked to the national database   - Pharmacists who compile SACT data are not thinking about the use this has at national level, they only have in mind the prescription they are making for patients – tracking is a secondary outcome of their workflow - SACT data could provide the number on how many patients were treated with one drug as monotherapy or in combination |
| System integration & funding^[[87]](#endnote-88)^ | - NHS uses Blueteq data for rebates – it is an invoicing system that happens to be a data collection system - NHS England can link Blueteq to their financing system which is where all claims flow through - Funded and managed by NHS England | - Data is updated every 2 months (previously every month) to reduce bureaucratic burden - Since hospital data systems are linked to the SACT, it reduces burden – physicians fill in information for prescription anyway, and SACT data collection is therefore a secondary phenomenon - SACT and Blueteq data can complement each other - Funded and managed by NHS England |
| Stakeholder involvement^[[88]](#endnote-89)^ | - NHS England and healthcare commissioners have access to the system | - Previously owned by Public Health England, then NHS Digital and now NHS England – it makes sense for NHS England to manage the SACT since they pay for oncology drugs |
| General comments^[[89]](#endnote-90)^ | - The current policy in England does not support pricing by indication, but the updated commercial framework specifies that commercial flexibility can be provided under certain circumstances   - Companies have the right to request commercial flexibility (and will have to pay for this privilege) if ICER < 20,000 - Uploading data to SACT is difficult when there are a variety of different prescribing systems between individual hospitals - Pharmacists who compile SACT data are not thinking about the use this has at national level, they only have in mind the prescription they are making for patients – tracking is a secondary outcome of their workflow - SACT and Blueteq data can complement each other - Algorithms may need to be developed in order to ensure the correct data is pulled for tracking – a pilot may need to be run to ensure this - The dataset landscapes in England are very comprehensive and complex, which leads to significant duplication - For companies with innovative products, Blueteq may be better than SACT as a source to negotiate rebates - Administrative burden can lead to reluctance to use data systems by physicians - One of the challenges with combinations is that ‘combinations are not always used in combination’ – one part of the combination has a finite maximum duration of treatment, but the other agent can continue to be used until disease progression means that they are not necessarily always being used simultaneously   - Therefore, appropriate coding by indication is needed | |

## France

Table 4. Hospital Discharge Data System (PMSI)

| Framework component | Details |
| --- | --- |
| Data source | - For both ambulatory and hospital medicines, data is recorded through hospital records, prescription registers, national cancer registers and National claims data – the SNDS (National Health Data System), managed by the CNAM (French NHS)^^[[90]](#endnote-91)^^   - There are more efforts to collect data in oncology on detailed use of medicines, compared to other areas - The Programme de médicalisation des systèmes d’information (PMSI) database is used to track hospital utilization – it is mainly used to measure the activity of the hospitals and for budget allocation^^[[91]](#endnote-92)^,^[[92]](#endnote-93)^^ |
| Scope | - Only expensive hospital drugs included in the *liste-en-sus* are tracked^^[[93]](#endnote-94)^,^[[94]](#endnote-95)^^ - The PMSI database includes ICD-10 codes, medical procedures performed during hospital stay, date of discharge and length of stay, DRGs, costly medicines/medical devices paid on top of DRG tariffs (*liste-en-sus*)^^[[95]](#endnote-96)^^ - In the PMSI, combinations can only be tracked if both drugs are hospital-use drugs and not retail (typically an IV drug)^^[[96]](#endnote-97)^^    - Even when a drug included in DRG is used in combination with an innovative drug but is included in the innovative drug’s label, tracking should be possible for this combination - The SNDS database includes PMSI and the primary care database, which covers retail/community pharmacies – the primary care database tracks utilization but does not include the indication (though there is data on outpatient oncology products, e.g., oral drugs)^^[[97]](#endnote-98)^^ |
| Data granularity / completeness | - The SNDS contains individual-level data on treatment duration, safety (only in some instances, e.g., serious adverse events leading to hospitalization), effectiveness (only in some cases), expenditure and dispensing data^^[[98]](#endnote-99)^^   - For drugs in the liste-en-sus and thus captured through PMSI, the indication is recorded^^[[99]](#endnote-100)^^   - The primary care database does not track the indication, but reasonable assumptions can be made based on data under each unique patient identifier (i.e., diagnosed indication can be inferred from the hospital discharge diagnoses)^^[[100]](#endnote-101)^^ - If a patient is given an IV cancer drug in hospital, then receives an oral cancer drug dispensed from a retail/community pharmacy as an add-on (combination), there is no separate code that indicates that these were used in combination – however, the use in combination can be inferred based on timing of use of the 2 components on a per patient basis through their unique patient identifier^^[[101]](#endnote-102)^^   - Patient care is linked between primary and secondary care - The CIP code, a numeric identifier for pharmacy products, is used^^[[102]](#endnote-103)^^ |
| Data access / transparency | - The national SNDS claims data are officially publicly available. In practice, there is long and complex procedures to access the data^^[[103]](#endnote-104)^^   - A study proposal must be developed (e.g., the protocol), then 2 bodies will review this proposal and this will take around 4 months^^[[104]](#endnote-105)^^   - The proposal will then be sent to CNAM (the NHS) for data extraction from the full SNDS dataset of 60 million people, but this takes 4-6 months due to a limited number of people doing the extraction^^[[105]](#endnote-106)^^   - There is now a new dataset called the ENSD, which is a random 2% sample of the full dataset (this does not require CNAM extraction from the full SNDS dataset)^^[[106]](#endnote-107)^^ - Pharmaceutical companies can have direct access to PMSI data, but can also access the data via vendors – the data can be used for pricing and reimbursement purposes^^[[107]](#endnote-108)^^ - PMSI data is readily available to industry for a price (anyone can purchase PMSI data) – however, this is changing soon and will only be accessible for public health purposes^^[[108]](#endnote-109)^^   - Access to primary care data is limited and requires the full process of proposal and CNAM extraction - Irreversible pseudonymization of data reduces data privacy concerns^^[[109]](#endnote-110)^^ |
| Data quality | - There are some concerns on the quality of the data on diagnosis, since data on the indication has to be entered manually^^[[110]](#endnote-111)^^ - The recorded indication may not be very reliable due to the administrative burden (up to 30% of indications are wrong) – although there have been a decrease in the rate of errors, there are still errors^^[[111]](#endnote-112)^^ - All hospitals declare data every day or every month (depending on internal hospital policies), but pharmaceutical companies will only receive PMSI data in September the year after (6-9 months delay)^^[[112]](#endnote-113)^^ - PMSI data is updated annually in April/May, while the full SNDS database is updated and made available in September^^[[113]](#endnote-114)^^ |
| Data management | - Clinicians fill in information for liste-en-sus and specify the indication, which is linked to the PMSI^^[[114]](#endnote-115)^^ |
| System integration & funding | - The PMSI database is linked to the SNIIR-AM database and the CépiDc causes of death database to form the French administrative health care database (SNDS) – SNDS is part of the Health Data Hub (the Health Data Hub initiative aims to link existing health databases in France to facilitate their use)^^[[115]](#endnote-116)^^ - There is no linkage between medical records and reimbursement claims data, so medical records cannot be used easily to identify the indication – this is unless the medical records are linked to the SNDS^^[[116]](#endnote-117)^^ - The databases are funded nationally^^[[117]](#endnote-118)^^ |
| Stakeholder involvement | - The PMSI is managed by the national health insurance authority^^[[118]](#endnote-119)^^ |
| General comments^[[119]](#endnote-120)^ | - PMSI data can be used for pricing and reimbursement negotiations, especially since the data is considered ‘neutral’ compared to disputable data from vendors like IQVIA - The government may wish to establish a tracking system to ensure physicians are using therapies appropriately to reduce off-label use - Data should not be used for marketing (to increase use of a specific drug) and insurance (discrimination for insurance coverage) purposes |

## Italy

Table 5. AIFA Monitoring Registries

| Framework component | Details |
| --- | --- |
| Data source | - The AIFA monitoring (product) registries are based on administrative data, launched at a national level as a consequence of pricing and reimbursement decisions – launched primarily for appropriateness of use, but were then used to implement MEA (but drugs can have registers without being under MEA)^^[[120]](#endnote-121)^,^[[121]](#endnote-122)^^   - AIFA registries mainly serve drugs whose therapeutic indications have been recognized as innovative   - For the other drugs (non-innovative drugs / indications), the registries serve as tools during price negotiation and to monitor prescription appropriateness (i.e., avoid off-label use)^[[122]](#endnote-123),^^[[123]](#endnote-124)^ - The register acts like a physician prescription request form, and also defines a maximum term of treatment duration^^[[124]](#endnote-125)^^   - E.g., for some drugs, there are some indications where they must stop at 24 months since the drug no longer reimbursed after 24 months - The complex regional healthcare system and financing means that claims data cannot be used – there is no national claims database, only regional^^[[125]](#endnote-126)^^ - AIFA has moved towards more financial-based agreements since 2018 (budget cap, price volume) instead of outcome-based agreements (payment by results) since it is simpler to use the registry just to assess appropriateness instead of outcomes^^[[126]](#endnote-127)^^ |
| Scope | - Frequently used only for innovative drugs (based on the AIFA innovation status framework launched in 2017) – both ‘fully innovative’ and ‘potentially innovative’ drugs^^[[127]](#endnote-128)^^   - However, there are exceptions – some drugs that are not innovative also have registries to handle clinical uncertainty   - Currently, there are around 200 drugs that are innovative/potentially innovative which have an AIFA registry, leading to a huge volume of data   - Around 90% of cancer drugs fall into the fully innovative / potentially innovative categories - The registries capture both monotherapies and combination therapies, as well as the indications^^[[128]](#endnote-129)^^ - Hospital drugs are usually tracked, but retail drugs are usually not^^[[129]](#endnote-130)^^   - If a combination therapy involves a hospital and a retail drug, this combination may not be tracked properly   - Some drugs which are Class A PHT have also been excluded from tracking since they involve retail pharmacies, though retail drugs can have their own registries |
| Data granularity / completeness | - Data is recorded by product (instead of by patient), and includes the diagnosis, indication, dose, line of treatment, number of packs/vials, previous treatment (including compassionate use), treatment duration, dispensing^^[[130]](#endnote-131)^,^[[131]](#endnote-132)^^   - The system automatically generates a dispensing form for pharmacies based on age   - The number of packs and the number of vials is required for pricing considerations - Data coverage is considered high due to implications for NHS reimbursement and data collection is mandated by law^^[[132]](#endnote-133)^^ |
| Data access / transparency | - Companies have access to data whenever they wish, but only have access to aggregated data from the registries (5-page report), with no efficacy data^^[[133]](#endnote-134)^,^[[134]](#endnote-135)^^   - Companies will only see the number of patients treated, amount of payback, number of patients per region and number of patients per indication   - Aggregated is defined as data aggregated to the hospital level - In the ideal setting, it may be good for pharmaceutical companies to have access to the same amount of data AIFA has access to in order to ensure re-negotiations run more smoothly (re-negotiations happen every 2 years)^^[[135]](#endnote-136)^^ - Technical guidance published by AIFA supports healthcare professionals (e.g., doctors, pharmacists), government stakeholders, and pharmaceutical companies to access the registers and appropriately use the registries^^[[136]](#endnote-137)^^ |
| Data quality | - Data quality is an issue as there is no auditing program – for example, a drug may be used for 7-8 months, but only 3 months of use is actually captured^^[[137]](#endnote-138)^,^[[138]](#endnote-139)^^   - This can be due to the fact that if payment by results is at 3 months, then pharmacists might not record the data for the 4^th^ month because it does not directly impact reimbursement   - When comparing packs sold tracked through the register and internal sales data, there is a 10-11% discrepancy - The high administrative burden of the registry means that physicians do not fill in the data even if they are obliged by law, and there is a lag in data entry (6 months to 1 year)^^[[139]](#endnote-140)^^   - However, the lag is not unreasonable for drugs under MEA since data entry needs to be timely for reimbursement |
| Data management | - The registries are governed and managed by AIFA^^[[140]](#endnote-141)^^ - AIFA has separate agreements with different companies for a novel combination therapy – i.e., each drug will have its own registry as there are separate volume-price agreements for 2 different molecules^^[[141]](#endnote-142)^^   - This depends on whether the drugs are used simultaneously or 1 after the other, and if the drugs are from the same company / 2 separate companies^^[[142]](#endnote-143)^^   - Setting up these registries requires quite a bit of work via the IT system: they need to be designed to permit industry to have access only to their own data   - The registry enables products to have different prices across different indications^^[[143]](#endnote-144)^^ - The registries have standardized, computerized procedures for each treatment phase (e.g., patient eligibility, supply, dispensing, follow-up)^^[[144]](#endnote-145)^^   - The prescription form includes monotherapy or combination therapy based on the label indication, then the system automatically provides dispensing instructions to the pharmacist – this strict control is implemented to avoid off-label use^^[[145]](#endnote-146)^^   - For the payback system (in MEAs), there is an algorithm that automatically creates the request for the pharmaceutical company, and the % of errors is very low^^[[146]](#endnote-147)^^ - Unique electronic patient demographic e-form enables linkage of records by patient across several drug registries – this allows analysis of populations treated with drug in dissimilar therapeutic areas^^[[147]](#endnote-148)^^ |
| System integration & funding | - The AIFA registry is a closed, separate system from local hospital systems, which means that physicians have to enter data twice – once for into the hospital records and once for the AIFA registry^^[[148]](#endnote-149)^^   - The administrative burden on physicians is a huge concern – there needs to be more people employed within AIFA and in hospitals to handle the registries   - This is different from Valtermed in Spain, since Valtermed can theoretically use hospital data - It is complex to maintain this registry in the long term – the administrative burden makes things difficult^^[[149]](#endnote-150)^,^[[150]](#endnote-151)^^ - AIFA registries are managed by AIFA, but is funded by pharmaceutical companies^^[[151]](#endnote-152)^^   - Funds from the marketing authorization holder are allocated mainly to construct data collection forms, updates on data collection and aggregated standard reports; data processing is funded by AIFA^^[[152]](#endnote-153)^^ |
| Stakeholder involvement | - AIFA manages the AIFA registries^^[[153]](#endnote-154)^^ - For a company that is involved in a combination therapy, they can potentially use registry data to show added value in re-negotiations^^[[154]](#endnote-155)^^ - To prescribe a drug, physicians must fill in the register for each patient^^[[155]](#endnote-156)^^   - Each registry allows the purchase and utilization of the drug only if a specific patient record/form is filled, then transmitted to the hospital pharmacist, to AIFA, and to the manufacturer – therefore, the data is easily traceable^^[[156]](#endnote-157)^^ - Information included in the registries is decided by the scientific committee of AIFA – no patient organizations are involved^^[[157]](#endnote-158)^^   - External experts are involved, e.g., for cancer drugs, there are close collaborations with oncologist associations - Companies are also involved in the registry discussion as they can propose things to AIFA scientific committee (CTS)^^[[158]](#endnote-159)^^ |
| General comments | - Italy has no clear strategy on combination therapies – AIFA would not necessarily support combination-specific pricing - Future considerations:   - Public-private partnerships to collect more observational data in collaboration with industry, and to reduce administrative burden   - Registry data can be used to validate companies’ internal data   - Tracking through EHR is not feasible yet   - Countries need to identify a source of data for tracking, whether it is an existing database or if a new database is needed |

## Spain

Table 6. Catalan Regional Health Data System and National Valtermed Data System

| Framework component | Details on Valtermed | Details on the Catalan Regional Health Data System |
| --- | --- | --- |
| Data source^[[159]](#endnote-160)^ | - Valtermed is a national registry used to monitor prescriptions of high-cost drugs with high clinical uncertainty under managed entry agreements - Since the ministry is not involved in invoicing, completion of Valtermed is completely ‘voluntary’, even if the ministry of health says it is mandatory for physicians/hospitals to fulfil data, there are no consequences if they do not fill in data | - The Catalan registry is a regional registry covering most hospitals in Catalonia that allows the Catalan Health Service to validate hospital invoices (if the hospital invoicing is aligned with the data from registry, they would reimburse the drug; if the hospital data is incomplete or does not match the registry data, then the reimbursement is not processed) - Catalonia re-designed electronic systems to benefit a number of situations / purposes - There are similar systems in Valencia and Madrid |
| Scope^[[160]](#endnote-161)^ | - Valtermed is currently used for high-cost medicines that have high clinical uncertainty (e.g., rare disease drugs) | - The Catalan registry covers outpatient drugs dispensed from hospitals (includes day hospital drugs like oncology drugs)   - Inpatient drugs like chemotherapy agents are not tracked, but if the chemotherapy is used in combination with an outpatient drug (e.g., immunotherapy), then it can be tracked (but without detailed data, e.g., data on dose and number of units used) - Success is due to the linkage between invoicing from healthcare facilities – there is high incentive for physicians and pharmacists to enter data in order to obtain reimbursement   - Not currently fully linked, but expected to be in 2025 |
| Data granularity / completeness^[[161]](#endnote-162)^ | - Valtermed captures the use of drugs per indication | - Data is collected by product instead of by patient - Both prescription data and invoicing data are captured, where prescription data contains clinical information, while invoicing data contains the number of units used - Individual-level data on patients, diagnosis, indication, number of units per month, treatment duration, effectiveness and safety are collected - There is a unique patient identifier – this allows linkage between prescription data containing clinical information and invoicing data that contains the number of units that has been used |
| Data access / transparency^[[162]](#endnote-163)^ | - Data cannot be accessed for commercial purposes | - Catalan links medical records to invoicing data, so data might not be fully anonymous - Data cannot be accessed for commercial purposes - Aggregated data is defined to be data aggregated at the regional level (17 regions) |
| Data quality^[[163]](#endnote-164)^ | - Since Valtermed is not linked to invoicing, there is no pressure for physicians/hospitals to fulfil data requirements, so the reporting rate is low - Half of the data that is in Valtermed is from Catalan region, and they represent only 20-25% of Spain | - There are systems in place for data quality checks and in general, the data is considered good quality (~80% accurate)   - The main problem was the date treatment is stopped as this information is not linked with reimbursement - Data is checked automatically between the patient medical records and the hospital invoices - Catalan Health Service is a contracting type of health provider – as part of the contract, each hospital site is required to collect data and can be inspected at any time |
| Data management^[[164]](#endnote-165)^ | - Physicians are expected to input data into Valtermed, but lack incentive | - The Catalan registry looks like an electronic patient record to physicians – it is automatically filled when a physician makes a prescription   - Some sites work with closed prescription protocols by indication that only allow some choices – deviations from the pre-specified list requires specific approval by the pharmacy - Combination therapies are captured through this system at the patient-level – each component of the combination treatment is collected as single treatment and they can be identified when used in combination   - At the time of invoicing, each component is separate and health service pays for each component |
| System integration & funding | - Standalone system coordinated by the Ministry of Health^^[[165]](#endnote-166)^^ - Regions like Catalan export their own registry data into Valtermed^^[[166]](#endnote-167)^^ - Nationally funded^^[[167]](#endnote-168)^^ | - Catalan exports registry data into Valtermed^^[[168]](#endnote-169)^^ - The Catalan registry is linked to the hospital invoicing system^^[[169]](#endnote-170)^^ - Funded by the Catalan Health Service^^[[170]](#endnote-171)^,^[[171]](#endnote-172)^^ |
| Stakeholder involvement | - Centrally managed by the Ministry of Health^[[172]](#endnote-173)^ | - Managed by the Catalan Health Service^^[[173]](#endnote-174)^^ |
| General comments^[[174]](#endnote-175)^ | - There are significant differences in terms of data infrastructure in Spain due to regional organization of health services - Specific initiatives on data infrastructure from Valencia, Catalonia and Madrid were the seed for the proposal of Valtermed - The system in the Catalan region is quite similar to Australia, in the sense that in the sense that it is instrumental for invoicing   - There is a requirement for pharmacists to collect information on indications, clinical data and follow ups in order to get the reimbursement   - However, the prescription system is separate for each of the 68 hospitals in Catalan, which means data is siloed – Catalan is trying to standardize the invoicing system between the 68 hospitals to make it interoperable by 2025   - The aim is to have a single database that covers all drugs (inpatient, outpatient, retail and hospital) - The infrastructure to implement combination-specific pricing exists in the Catalan region, but drug prices are determined at the national level – price is set per unit and is the same across all indications   - Every time when a new indication or combination appears the price is usually decreasing because there is no way to distinguish the used per indication at the national level   - In the scenario where the price would be determined at the regional level, then Catalan region with its system would be able know for how many patients per indication a product was used for, and whether it was used in combination - Reimbursement/claims data is the best option for tracking – the main point is to complement this data with the minimal amount of data that is needed from a registry or EHR, such as the indication if this is not captured in the claims data (if there are no separate reimbursement codes for different indications) - Data collection should be practical, reduce duplicative data entry by syncing to other data systems, and standardized so it can able aggregated across regions - Aggregated data can be used to negotiate with industry - To gain support from physicians, it is important to show them how data is used (e.g., saving budgets), and for them to be able to use the data to publish studies. Benchmarks can be created to increase competitiveness between hospitals on data capture | |

## Sweden

Table 7. National Quality Registries for Cancer on the Information Network for Cancer care (INCA), supported by Regional Care Centers (RCC)

| Framework component | Details |
| --- | --- |
| Data source | - Sweden can only track outpatient products (retail pharmacies), which means oncology products are not captured, and mainly for academic purposes (not for pricing and reimbursement)^^[[175]](#endnote-176)^,^[[176]](#endnote-177)^^ - Cancer registries are therefore the only current option that can facilitate tracking of hospital oncology products^^[[177]](#endnote-178)^^ |
| Scope | - Cancer registries cover a range of cancers^^[[178]](#endnote-179)^^ - In theory, the registries are national in scope, but actual coverage is very low^^[[179]](#endnote-180)^^ - Other databases currently only cover outpatient medicines^^[[180]](#endnote-181)^^ |
| Data granularity / completeness | - Data captured in the cancer registries is relatively granular, but the poor completeness of data reduces the utility^[[181]](#endnote-182)^ |
| Data access / transparency | - The registries are run by regional cancer centers and access is quite ungoverned^[[182]](#endnote-183)^ |
| Data quality | - The coverage rate is very low in the cancer registries since they are run on a voluntary basis – there is no systematic collection of data, leading to low reporting rate from physicians^^[[183]](#endnote-184)^^ - A 1-year delay in data is not expected to be a massive issue since rebates are paid annually anyway^^[[184]](#endnote-185)^^ |
| Data management | - Currently in hospitals, physicians are not obliged to add the indication or diagnosis when they request medicines – as such, there is no way to track whether a medicine is used in combination or as monotherapy (even if there was a reimbursement claims database)^^[[185]](#endnote-186)^^ - Physicians choose to enter data into the cancer registries, but they lack the incentive to^^[[186]](#endnote-187)^^ |
| System integration & funding | - Cancer registries are independent from existing data systems, which increases administrative burden for physicians (and therefore leads to low completeness)^^[[187]](#endnote-188)^^ - It should be noted that almost all databases in Sweden use the same patient identifier, so it is theoretically quite easy to link data collected in national databases^^[[188]](#endnote-189)^^ - The registries are maintained by the regional cancer centers, which in turn are funded by the regions^^[[189]](#endnote-190)^^ |
| Stakeholder involvement | - The registries are run by regional cancer centers^[[190]](#endnote-191)^ |
| General comments^[[191]](#endnote-192)^ | - Hospital pharmacies do not track inpatient medicines at the patient-level – it is captured in medical records, but these are difficult to access   - Sweden has a decentralized healthcare system with 21 regions, and there is no joint system for electronic medical records – regions and even individual hospitals will use different systems, so the interoperability of databases is extremely low   - Therefore, tracking on a national level based on medical records would be very difficult - There has been some work in developing a common infrastructure for such healthcare issues, e.g., the National Service Platform   - It is supposed to be able to tap into the different systems in the different regions to look at certain questions – but it is only searchable by the personal identification number and not therapies (e.g., combination therapies), so it is not really a feasible source - The National Medication List has been introduced, but only for outpatient drugs, managed by the Swedish e-Health Agency, but this has not been integrated into the regions yet (due to difficulty in merging regional data systems with this national database)   - Some infrastructure investment would be needed to make the National Medication List applicable for inpatient drugs as well   - It is expected for the National Medication List system to be integrated into existing systems (e-prescription, EHRs) - Pre-authorization does not exist for oncology products - Registries’ coverage is too low to be used for tracking at the moment – the coverage will need to be improved by improving the practices of physicians   - The problem with registries is that the physicians cannot be bothered to enter the data – the physicians do not feel that they get that much out of such a system, and it is not ‘worth it’ for them   - Even if financial incentives are provided for data entry, that financial incentive is going to end up with the clinic or the region anyway and not back to the physician / hospital so there is very little incentive to enter the data - Tracking should be a secondary outcome of clinicians’ workflow – clinicians will provide prescriptions and tracking will be a secondary benefit   - To gain support from physicians for such a system, it has to be integrated into existing EHR systems to reduce administrative burden and the process has to be automated - Tracking utilization of hospital products in general has many more benefits than just pricing – e.g., use appropriateness, safety monitoring, RWE studies, etc. – and therefore it should be done irrespective of pricing and reimbursement considerations - Swedish payers do not have a big appetite for outcomes-based agreements mainly because follow-up is cumbersome and there is a lot of administration and is very expensive - Overall, data should be aggregated at the level of the payer / authority that is in charge of pricing and reimbursement, whether it is national or regional |

## Switzerland

Table 8. SmartMIP Data System

| Framework component | Details |
| --- | --- |
| Data source | - SmartMIP is a third-party software that hosts data from sick funds for pharmaceutical companies to access – it acts as an interface between sick funds and pharmaceutical companies for the processing of payment reclaims , - The infrastructure for indication-based pricing (IBP) has been developed – all reimbursed indications now have an indication code   - IBP has been previously implemented, suggesting that tracking utilization is possible – Lenalidomide (Revlimid®) has different prices when used in combination with carfilzomib (Kyprolis®) or elotuzumab (Empliciti®) |
| Scope | - Drugs included in Article 71 and price models for medicines in the list of specialty medicines, which includes oncology combination products^^[[192]](#endnote-193)^^ - Pharmaceutical companies decide which products they would like to include in SmartMIP – they will only include drugs and indications that have a price model since SmartMIP is the easiest system to manage reclaims, so there is therefore no overview of utilization across all indications of one drug^^[[193]](#endnote-194)^^ |
| Data granularity / completeness | - The indication code used is detailed, and includes prescription restrictions as well as combination use, but health insurances do not release data by indication^^[[194]](#endnote-195)^^   - Currently, there are discussions about putting the indication code on the invoice, so that there is a link between the product that was used, and what it is used for – however, this is very controversial - Patients do not have a single unique identifier, so it is difficult to track utilization if patients switch funds or if the funds decide to change their numbering system^^[[195]](#endnote-196)^^ - Start date of use and end date of use is not captured^^[[196]](#endnote-197)^^ |
| Data access / transparency | - Industry can access SmartMIP data on their own products (including combinations) – there is restricted visibility dependent on need, since sensitive and confidential patient data is not shared with everyone^^[[197]](#endnote-198)^^ - The national identifier cannot be used due to data privacy concerns^^[[198]](#endnote-199)^^ - There is no consensus on the benefit of increasing data transparency^^[[199]](#endnote-200)^^   - Payers are in between, pharmaceutical companies are pro, while patient organizations, some politicians and some health insurance associations are against it |
| Data quality | - Approximately 90% of sick funds send data through SmartMIP – it is not 100% because it is not mandated by the government^^[[200]](#endnote-201)^^ - Doctors provide the indication code to sick funds, but sick funds mostly do not report the codes accurately to pharmaceutical companies since they do not officially record the indication^^[[201]](#endnote-202)^^ - Data quality in SmartMIP is significantly limited by the quality of data obtained from sick funds – reimbursement mistakes are over 10%^^[[202]](#endnote-203)^^   - Physicians can choose the wrong code, even if they can be penalized by insurance funds for this – sometimes a drug is recorded under both Article 71 and a pricing model (which is not allowed) for one patient   - Algorithms have to be used to compensate for poor data quality, and SmartMIP also runs validation checks on the data; pharmaceutical companies also have to manually correct data themselves - Data quality varies between sick funds since the data systems are not standardized^^[[203]](#endnote-204)^^ - Data is subject to time lag since physicians can take 2 months to file invoices, and sick funds then take time to process the invoice (can take 2 years to make claims) – pharmaceutical companies usually receive data with around a 5-month delay^^[[204]](#endnote-205)^^ |
| Data management | - Data collection is enabled by an indication code being noted on each prescription at the hospital level – the health service provider sends a cost coverage request to the sick fund, who then responds to the request for reimbursement^^[[205]](#endnote-206)^,^[[206]](#endnote-207)^^   - However, once the indication code is submitted to the sick funds, only the medical examiner and a few people in the sick funds can see the indication (the medical examiner) due to data privacy reasons, and the indication is then buried somewhere (not shared with SmartMIP)   - Health insurances do not release data by indication – SmartMIP has written software that matches data from the medical examiner and the invoice, to generate the claims - The cost coverage requests can be handwritten and sent by mail or fax – only recently introduced electronic cost coverages^^[[207]](#endnote-208)^^   - Hospitals will have different electronic systems, and it will take years to implement a homogeneous electronic cost coverage system |
| System integration & funding | - Sick funds use SmartMIP for reclaims, but it is not integrated into the national health system^^[[208]](#endnote-209)^^ - SmartMIP is completely paid for by industry^^[[209]](#endnote-210)^^ - There are discussions on asking sick funds to start paying for data / tracking, but they are unenthusiastic about this prospect^^[[210]](#endnote-211)^^ |
| Stakeholder involvement | - There is no federal data custodian – a national health body that aggregates and standardizes data is needed^^[[211]](#endnote-212)^^ - SmartMIP is developed by a third-party vendor, Aquantic AG^^[[212]](#endnote-213)^^ - SmartMIP would benefit significantly if there was government support – data entry can be standardized if the government sets out official guidance (currently, the ~50 sick funds record data differently)^^[[213]](#endnote-214)^^   - Lack of support from government authorities leads to inconsistent data collection – due to the federated health system with multiple health funds, it is difficult to drive coordination without government involvement |
| General comments | - Currently, there are discussions about putting the indication code on the invoice, so that there is a link between the product that was used, and what it is used for – however, this is very controversial - There is no consensus between health insurers, pharmaceutical companies, patient organisations and politicians on the benefit of increasing data transparency - SmartMIP reimbursement claims data can be used to prove inefficiencies in health insurance coverages to save money - If the government mandates proper data entry and tracking, then the situation in Switzerland will potentially change |

# References

1. Sturkenboom, M., & Schink, T. (Eds.). (2021). Databases for Pharmacoepidemiological Research. Springer Series on Epidemiology and Public Health. doi:10.1007/978-3-030-51455-6 [↑](#endnote-ref-2)
2. <https://www.aihw.gov.au/about-our-data/our-data-collections/pharmaceutical-benefits-scheme> [↑](#endnote-ref-3)
3. Australia expert interview [↑](#endnote-ref-4)
4. Australia expert interview [↑](#endnote-ref-5)
5. OECD. (2019). Using routinely collected data to inform pharmaceutical policies - OECD. https://www.oecd.org/health/health-systems/Using-Routinely-Collected-Data-to-Inform-Pharmaceutical-Policies-Analytical-Report-2019.pdf [↑](#endnote-ref-6)
6. OECD. (2019). Pharmaceutical Expenditure Tracking Budgeting Forecasting Country notes. https://www.oecd.org/health/health-systems/Pharmaceutical-Expenditure-Tracking-Budgeting-Forecasting-Country-Notes.pdf [↑](#endnote-ref-7)
7. Sturkenboom, M., & Schink, T. (Eds.). (2021). Databases for Pharmacoepidemiological Research. Springer Series on Epidemiology and Public Health. doi:10.1007/978-3-030-51455-6 [↑](#endnote-ref-8)
8. Sturkenboom, M., & Schink, T. (Eds.). (2021). Databases for Pharmacoepidemiological Research. Springer Series on Epidemiology and Public Health. doi:10.1007/978-3-030-51455-6 [↑](#endnote-ref-9)
9. Australia expert interview [↑](#endnote-ref-10)
10. Mellish, L., Karanges, E. A., Litchfield, M. J., Schaffer, A. L., Blanch, B., Daniels, B. J., Segrave, A., &amp; Pearson, S.-A. (2015). The Australian Pharmaceutical Benefits Scheme Data Collection: A practical guide for researchers. BMC Research Notes, 8(1). https://doi.org/10.1186/s13104-015-1616-8 [↑](#endnote-ref-11)
11. Australia expert interview [↑](#endnote-ref-12)
12. Sturkenboom, M., & Schink, T. (Eds.). (2021). Databases for Pharmacoepidemiological Research. Springer Series on Epidemiology and Public Health. doi:10.1007/978-3-030-51455-6 [↑](#endnote-ref-13)
13. Australia expert interview [↑](#endnote-ref-14)
14. Australia expert interview [↑](#endnote-ref-15)
15. Australia expert interview [↑](#endnote-ref-16)
16. Australia expert interview [↑](#endnote-ref-17)
17. Australia expert interview [↑](#endnote-ref-18)
18. Australia expert interview [↑](#endnote-ref-19)
19. Australia expert interview [↑](#endnote-ref-20)
20. OECD. (2019). Using routinely collected data to inform pharmaceutical policies - OECD. https://www.oecd.org/health/health-systems/Using-Routinely-Collected-Data-to-Inform-Pharmaceutical-Policies-Analytical-Report-2019.pdf [↑](#endnote-ref-21)
21. Australia expert interview [↑](#endnote-ref-22)
22. Australia expert interview [↑](#endnote-ref-23)
23. Sturkenboom, M., & Schink, T. (Eds.). (2021). Databases for Pharmacoepidemiological Research. Springer Series on Epidemiology and Public Health. doi:10.1007/978-3-030-51455-6 [↑](#endnote-ref-24)
24. Sturkenboom, M., & Schink, T. (Eds.). (2021). Databases for Pharmacoepidemiological Research. Springer Series on Epidemiology and Public Health. doi:10.1007/978-3-030-51455-6 [↑](#endnote-ref-25)
25. Sturkenboom, M., & Schink, T. (Eds.). (2021). Databases for Pharmacoepidemiological Research. Springer Series on Epidemiology and Public Health. doi:10.1007/978-3-030-51455-6 [↑](#endnote-ref-26)
26. Australia expert interview [↑](#endnote-ref-27)
27. Australia expert interview [↑](#endnote-ref-28)
28. Australia expert interview [↑](#endnote-ref-29)
29. Australia expert interview [↑](#endnote-ref-30)
30. Australia expert interview [↑](#endnote-ref-31)
31. Australia expert interview [↑](#endnote-ref-32)
32. Australia expert interview [↑](#endnote-ref-33)
33. Sturkenboom, M., & Schink, T. (Eds.). (2021). Databases for Pharmacoepidemiological Research. Springer Series on Epidemiology and Public Health. doi:10.1007/978-3-030-51455-6 [↑](#endnote-ref-34)
34. Interview with local expert [↑](#endnote-ref-35)
35. Sturkenboom, M., & Schink, T. (Eds.). (2021). Databases for Pharmacoepidemiological Research. Springer Series on Epidemiology and Public Health. doi:10.1007/978-3-030-51455-6 [↑](#endnote-ref-36)
36. OECD. (2019). Using routinely collected data to inform pharmaceutical policies - OECD. https://www.oecd.org/health/health-systems/Using-Routinely-Collected-Data-to-Inform-Pharmaceutical-Policies-Analytical-Report-2019.pdf [↑](#endnote-ref-37)
37. Australia expert interview [↑](#endnote-ref-38)
38. https://www.ehealth.fgov.be/fr/professionnels-de-la-sante/services/civars-chapitre-iv-et-viii [↑](#endnote-ref-39)
39. https://www.ima-aim.be/-Donnees-de-sante-?lang=fr [↑](#endnote-ref-40)
40. https://www.ima-aim.be/-Donnees-de-sante-?lang=fr [↑](#endnote-ref-41)
41. OECD. (2019). Using routinely collected data to inform pharmaceutical policies - OECD. https://www.oecd.org/health/health-systems/Using-Routinely-Collected-Data-to-Inform-Pharmaceutical-Policies-Analytical-Report-2019.pdf [↑](#endnote-ref-42)
42. Belgium expert interview [↑](#endnote-ref-43)
43. Belgium expert interview [↑](#endnote-ref-44)
44. Belgium expert interview [↑](#endnote-ref-45)
45. Belgium expert interview [↑](#endnote-ref-46)
46. Belgium expert interview [↑](#endnote-ref-47)
47. Belgium expert interview [↑](#endnote-ref-48)
48. Belgium expert interview [↑](#endnote-ref-49)
49. Belgium expert interview [↑](#endnote-ref-50)
50. Belgium expert interview [↑](#endnote-ref-51)
51. Belgium expert interview [↑](#endnote-ref-52)
52. Belgium expert interview [↑](#endnote-ref-53)
53. Belgium expert interview [↑](#endnote-ref-54)
54. Belgium expert interview [↑](#endnote-ref-55)
55. Belgium expert interview [↑](#endnote-ref-56)
56. Belgium expert interview [↑](#endnote-ref-57)
57. Belgium expert interview [↑](#endnote-ref-58)
58. Belgium expert interview [↑](#endnote-ref-59)
59. Belgium expert interview [↑](#endnote-ref-60)
60. Belgium expert interview [↑](#endnote-ref-61)
61. Belgium expert interview [↑](#endnote-ref-62)
62. Belgium expert interview [↑](#endnote-ref-63)
63. Belgium expert interview [↑](#endnote-ref-64)
64. Belgium expert interview [↑](#endnote-ref-65)
65. Belgium expert interview [↑](#endnote-ref-66)
66. Belgium expert interview [↑](#endnote-ref-67)
67. Belgium expert interview [↑](#endnote-ref-68)
68. Belgium expert interview [↑](#endnote-ref-69)
69. Belgium expert interview [↑](#endnote-ref-70)
70. https://www.blueteq.com/latestnews/ArticleID/2/Blueteq-established-as-the-system-for-High-Cost-Drugs-Management-Process-in-NHS-Englands-Commissioning-Intentions-2016-2017.html [↑](#endnote-ref-71)
71. https://www.ardengemcsu.nhs.uk/showcase/case-studies/case-studies/developing-an-online-high-cost-drug-approval-system/ [↑](#endnote-ref-72)
72. https://www.gilliankenny.com/blog/what-is-the-blueteq-high-cost-drug-management-system/ [↑](#endnote-ref-73)
73. England expert interview [↑](#endnote-ref-74)
74. England expert interview [↑](#endnote-ref-75)
75. England expert interview [↑](#endnote-ref-76)
76. England expert interview [↑](#endnote-ref-77)
77. England expert interview [↑](#endnote-ref-78)
78. England expert interview [↑](#endnote-ref-79)
79. England expert interview [↑](#endnote-ref-80)
80. England expert interview [↑](#endnote-ref-81)
81. England expert interview [↑](#endnote-ref-82)
82. England expert interview [↑](#endnote-ref-83)
83. England expert interview [↑](#endnote-ref-84)
84. England expert interview [↑](#endnote-ref-85)
85. England expert interview [↑](#endnote-ref-86)
86. England expert interview [↑](#endnote-ref-87)
87. England expert interview [↑](#endnote-ref-88)
88. England expert interview [↑](#endnote-ref-89)
89. England expert interview [↑](#endnote-ref-90)
90. Pisana A, Wettermark B, Kurdi A, Tubic B, Pontes C, Zara C, Van Ganse E, Petrova G, Mardare I, Fürst J, Roig-Izquierdo M, Melien O, Bonanno PV, Banzi R, Marković-Peković V, Mitkova Z and Godman B (2022) Challenges and Opportunities With Routinely Collected Data on the Utilization of Cancer Medicines. Perspectives From Health Authority Personnel Across 18 European Countries. Front. Pharmacol. 13:873556. doi: 10.3389/fphar.2022.873556 [↑](#endnote-ref-91)
91. OECD. (2019). Using routinely collected data to inform pharmaceutical policies - OECD. https://www.oecd.org/health/health-systems/Using-Routinely-Collected-Data-to-Inform-Pharmaceutical-Policies-Analytical-Report-2019.pdf [↑](#endnote-ref-92)
92. France expert interview https://fr.wikipedia.org/wiki/Programme_de_m%C3%A9dicalisation_des_syst%C3%A8mes_d%27information [↑](#endnote-ref-93)
93. https://www.iqvia.com/-/media/iqvia/pdfs/isporbarcelona2018posters/november-13/one-product-different-indicationsdifferent-prices.pdf [↑](#endnote-ref-94)
94. France expert interview [↑](#endnote-ref-95)
95. OECD. (2019). Using routinely collected data to inform pharmaceutical policies - OECD. https://www.oecd.org/health/health-systems/Using-Routinely-Collected-Data-to-Inform-Pharmaceutical-Policies-Analytical-Report-2019.pdf [↑](#endnote-ref-96)
96. France expert interview [↑](#endnote-ref-97)
97. France expert interview [↑](#endnote-ref-98)
98. Pisana A, Wettermark B, Kurdi A, Tubic B, Pontes C, Zara C, Van Ganse E, Petrova G, Mardare I, Fürst J, Roig-Izquierdo M, Melien O, Bonanno PV, Banzi R, Marković-Peković V, Mitkova Z and Godman B (2022) Challenges and Opportunities With Routinely Collected Data on the Utilization of Cancer Medicines. Perspectives From Health Authority Personnel Across 18 European Countries. Front. Pharmacol. 13:873556. doi: 10.3389/fphar.2022.873556 [↑](#endnote-ref-99)
99. France expert interview [↑](#endnote-ref-100)
100. France expert interview [↑](#endnote-ref-101)
101. France expert interview [↑](#endnote-ref-102)
102. France expert interview [↑](#endnote-ref-103)
103. Pisana A, Wettermark B, Kurdi A, Tubic B, Pontes C, Zara C, Van Ganse E, Petrova G, Mardare I, Fürst J, Roig-Izquierdo M, Melien O, Bonanno PV, Banzi R, Marković-Peković V, Mitkova Z and Godman B (2022) Challenges and Opportunities With Routinely Collected Data on the Utilization of Cancer Medicines. Perspectives From Health Authority Personnel Across 18 European Countries. Front. Pharmacol. 13:873556. doi: 10.3389/fphar.2022.873556 [↑](#endnote-ref-104)
104. France expert interview [↑](#endnote-ref-105)
105. France expert interview [↑](#endnote-ref-106)
106. Pisana A, Wettermark B, Kurdi A, Tubic B, Pontes C, Zara C, Van Ganse E, Petrova G, Mardare I, Fürst J, Roig-Izquierdo M, Melien O, Bonanno PV, Banzi R, Marković-Peković V, Mitkova Z and Godman B (2022) Challenges and Opportunities With Routinely Collected Data on the Utilization of Cancer Medicines. Perspectives From Health Authority Personnel Across 18 European Countries. Front. Pharmacol. 13:873556. doi: 10.3389/fphar.2022.873556 [↑](#endnote-ref-107)
107. France expert interview [↑](#endnote-ref-108)
108. France expert interview [↑](#endnote-ref-109)
109. France expert interview [↑](#endnote-ref-110)
110. Pisana A, Wettermark B, Kurdi A, Tubic B, Pontes C, Zara C, Van Ganse E, Petrova G, Mardare I, Fürst J, Roig-Izquierdo M, Melien O, Bonanno PV, Banzi R, Marković-Peković V, Mitkova Z and Godman B (2022) Challenges and Opportunities With Routinely Collected Data on the Utilization of Cancer Medicines. Perspectives From Health Authority Personnel Across 18 European Countries. Front. Pharmacol. 13:873556. doi: 10.3389/fphar.2022.873556 [↑](#endnote-ref-111)
111. France expert interview [↑](#endnote-ref-112)
112. France expert interview [↑](#endnote-ref-113)
113. France expert interview [↑](#endnote-ref-114)
114. France expert interview [↑](#endnote-ref-115)
115. OECD. (2019). Using routinely collected data to inform pharmaceutical policies - OECD. https://www.oecd.org/health/health-systems/Using-Routinely-Collected-Data-to-Inform-Pharmaceutical-Policies-Analytical-Report-2019.pdf [↑](#endnote-ref-116)
116. France expert interview [↑](#endnote-ref-117)
117. France expert interview [↑](#endnote-ref-118)
118. France expert interview [↑](#endnote-ref-119)
119. France expert interview [↑](#endnote-ref-120)
120. Italy expert interview [↑](#endnote-ref-121)
121. Italy expert interview [↑](#endnote-ref-122)
122. Xoxi E, Facey KM and Cicchetti A (2021) The Evolution of AIFA Registries to Support Managed Entry Agreements for Orphan Medicinal Products in Italy. Front. Pharmacol. 12:699466. doi: 10.3389/fphar.2021.699466 [↑](#endnote-ref-123)
123. Xoxi, E., Di Bidino, R., Leone, S., Aiello, A., & Prada, M. (2022). Value assessment of medicinal products by the Italian Medicines Agency (AIFA) and French National Authority for Health (HAS): Similarities and discrepancies. Frontiers in medical technology, 4, 917151. https://doi.org/10.3389/fmedt.2022.917151 [↑](#endnote-ref-124)
124. Italy expert interview [↑](#endnote-ref-125)
125. Italy expert interview [↑](#endnote-ref-126)
126. Italy expert interview [↑](#endnote-ref-127)
127. Italy expert interview [↑](#endnote-ref-128)
128. Italy expert interview [↑](#endnote-ref-129)
129. Italy expert interview [↑](#endnote-ref-130)
130. Italy expert interview [↑](#endnote-ref-131)
131. Italy expert interview [↑](#endnote-ref-132)
132. Italy expert interview [↑](#endnote-ref-133)
133. Italy expert interview [↑](#endnote-ref-134)
134. Italy expert interview [↑](#endnote-ref-135)
135. Italy expert interview [↑](#endnote-ref-136)
136. Italy expert interview [↑](#endnote-ref-137)
137. Italy expert interview [↑](#endnote-ref-138)
138. Italy expert interview [↑](#endnote-ref-139)
139. Italy expert interview [↑](#endnote-ref-140)
140. Italy expert interview [↑](#endnote-ref-141)
141. Italy expert interview [↑](#endnote-ref-142)
142. Italy expert interview [↑](#endnote-ref-143)
143. Italy expert interview [↑](#endnote-ref-144)
144. Italy expert interview [↑](#endnote-ref-145)
145. Italy expert interview [↑](#endnote-ref-146)
146. Italy expert interview [↑](#endnote-ref-147)
147. Xoxi E, Facey KM and Cicchetti A (2021) The Evolution of AIFA Registries to Support Managed Entry Agreements for Orphan Medicinal Products in Italy. Front. Pharmacol. 12:699466. doi: 10.3389/fphar.2021.699466 [↑](#endnote-ref-148)
148. Italy expert interview [↑](#endnote-ref-149)
149. Italy expert interview [↑](#endnote-ref-150)
150. Italy expert interview [↑](#endnote-ref-151)
151. Italy expert interview [↑](#endnote-ref-152)
152. Italy expert interview [↑](#endnote-ref-153)
153. Italy expert interview [↑](#endnote-ref-154)
154. Italy expert interview [↑](#endnote-ref-155)
155. Italy expert interview [↑](#endnote-ref-156)
156. Italy expert interview [↑](#endnote-ref-157)
157. Italy expert interview [↑](#endnote-ref-158)
158. Italy expert interview [↑](#endnote-ref-159)
159. Spain expert interview [↑](#endnote-ref-160)
160. Spain expert interview [↑](#endnote-ref-161)
161. Spain expert interview [↑](#endnote-ref-162)
162. Spain expert interview [↑](#endnote-ref-163)
163. Spain expert interview [↑](#endnote-ref-164)
164. Spain expert interview [↑](#endnote-ref-165)
165. <https://www.sanidad.gob.es/en/profesionales/farmacia/valtermed/home.htm> [↑](#endnote-ref-166)
166. Spain expert interview [↑](#endnote-ref-167)
167. Spain expert interview [↑](#endnote-ref-168)
168. Spain expert interview [↑](#endnote-ref-169)
169. Spain expert interview [↑](#endnote-ref-170)
170. Spain expert interview [↑](#endnote-ref-171)
171. Pisana A, Wettermark B, Kurdi A, Tubic B, Pontes C, Zara C, Van Ganse E, Petrova G, Mardare I, Fürst J, Roig-Izquierdo M, Melien O, Bonanno PV, Banzi R, Marković-Peković V, Mitkova Z and Godman B (2022) Challenges and Opportunities With Routinely Collected Data on the Utilization of Cancer Medicines. Perspectives From Health Authority Personnel Across 18 European Countries. Front. Pharmacol. 13:873556. doi: 10.3389/fphar.2022.873556 [↑](#endnote-ref-172)
172. Spain expert interview [↑](#endnote-ref-173)
173. Pisana A, Wettermark B, Kurdi A, Tubic B, Pontes C, Zara C, Van Ganse E, Petrova G, Mardare I, Fürst J, Roig-Izquierdo M, Melien O, Bonanno PV, Banzi R, Marković-Peković V, Mitkova Z and Godman B (2022) Challenges and Opportunities With Routinely Collected Data on the Utilization of Cancer Medicines. Perspectives From Health Authority Personnel Across 18 European Countries. Front. Pharmacol. 13:873556. doi: 10.3389/fphar.2022.873556 [↑](#endnote-ref-174)
174. Spain expert interview [↑](#endnote-ref-175)
175. Sweden expert interview [↑](#endnote-ref-176)
176. Pisana A, Wettermark B, Kurdi A, Tubic B, Pontes C, Zara C, Van Ganse E, Petrova G, Mardare I, Fürst J, Roig-Izquierdo M, Melien O, Bonanno PV, Banzi R, Marković-Peković V, Mitkova Z and Godman B (2022) Challenges and Opportunities With Routinely Collected Data on the Utilization of Cancer Medicines. Perspectives From Health Authority Personnel Across 18 European Countries. Front. Pharmacol. 13:873556. doi: 10.3389/fphar.2022.873556 [↑](#endnote-ref-177)
177. Sweden expert interview [↑](#endnote-ref-178)
178. Sweden expert interview [↑](#endnote-ref-179)
179. Sweden expert interview [↑](#endnote-ref-180)
180. Sweden expert interview [↑](#endnote-ref-181)
181. Sweden expert interview [↑](#endnote-ref-182)
182. Sweden expert interview [↑](#endnote-ref-183)
183. Sweden expert interview [↑](#endnote-ref-184)
184. Sweden expert interview [↑](#endnote-ref-185)
185. Sweden expert interview [↑](#endnote-ref-186)
186. Sweden expert interview [↑](#endnote-ref-187)
187. Sweden expert interview [↑](#endnote-ref-188)
188. Pisana A, Wettermark B, Kurdi A, Tubic B, Pontes C, Zara C, Van Ganse E, Petrova G, Mardare I, Fürst J, Roig-Izquierdo M, Melien O, Bonanno PV, Banzi R, Marković-Peković V, Mitkova Z and Godman B (2022) Challenges and Opportunities With Routinely Collected Data on the Utilization of Cancer Medicines. Perspectives From Health Authority Personnel Across 18 European Countries. Front. Pharmacol. 13:873556. doi: 10.3389/fphar.2022.873556 [↑](#endnote-ref-189)
189. Sweden expert interview [↑](#endnote-ref-190)
190. Sweden expert interview [↑](#endnote-ref-191)
191. Sweden expert interview [↑](#endnote-ref-192)
192. Switzerland expert interview [↑](#endnote-ref-193)
193. Switzerland expert interview [↑](#endnote-ref-194)
194. Switzerland expert interview [↑](#endnote-ref-195)
195. Switzerland expert interview [↑](#endnote-ref-196)
196. Switzerland expert interview [↑](#endnote-ref-197)
197. Switzerland expert interview [↑](#endnote-ref-198)
198. Switzerland expert interview [↑](#endnote-ref-199)
199. Switzerland expert interview [↑](#endnote-ref-200)
200. Switzerland expert interview [↑](#endnote-ref-201)
201. Switzerland expert interview [↑](#endnote-ref-202)
202. Switzerland expert interview [↑](#endnote-ref-203)
203. Switzerland expert interview [↑](#endnote-ref-204)
204. Switzerland expert interview [↑](#endnote-ref-205)
205. Switzerland expert interview [↑](#endnote-ref-206)
206. https://aquantic.ch/wp-content/uploads/2020/05/SmartMIP-Brosch%C3%BCre-2020-01-23.pdf [↑](#endnote-ref-207)
207. Switzerland expert interview [↑](#endnote-ref-208)
208. Switzerland expert interview [↑](#endnote-ref-209)
209. Switzerland expert interview [↑](#endnote-ref-210)
210. Switzerland expert interview [↑](#endnote-ref-211)
211. Switzerland expert interview [↑](#endnote-ref-212)
212. Switzerland expert interview [↑](#endnote-ref-213)
213. Switzerland expert interview [↑](#endnote-ref-214)
